# Epistatic interactions between at least three loci determine the “rat-tail” phenotype in cattle

**DOI:** 10.1186/s12711-016-0199-8

**Published:** 2016-03-31

**Authors:** Jacqueline Knaust, Frieder Hadlich, Rosemarie Weikard, Christa Kuehn

**Affiliations:** Institute for Genome Biology, Leibniz Institute for Farm Animal Biology, 18196 Dummerstorf, Mecklenburg-Vorpommern, Germany; Faculty of Agricultural and Environmental Sciences, University of Rostock, 18059 Rostock, Germany

## Abstract

**Background:**

The “rat-tail” syndrome (RTS) is an inherited hypotrichosis in cattle, which is exclusively expressed in diluted coloured hair. The affected animals also suffer from disturbed thermoregulation, which impairs their health and growth performance. Phenotypic features that are similar to RTS are observed in dogs with black hair follicle dysplasia.

**Results:**

We used a resource cross population between German Holstein and Charolais cattle breeds to prove that epistatic interactions between at least three independent genetic loci are required for the expression of the RTS phenotype. In this population, the RTS is exclusively expressed in animals with a eumelanic background that is due to the dominant *E*^D^ allele at the *melanocortin 1 receptor* gene located on *Bos taurus* autosome (BTA) 18. In addition, only the individuals that are heterozygous at the *dilution* locus on BTA5 that corresponds to the *premelanosome protein* or *silver* gene variant *c.64G*>*A* were classified as displaying a RTS phenotype. Linkage and whole-genome association analyses using different models and different pedigrees allowed us to map a third locus (hereafter referred to as the *RTS* locus) that is essential for the expression of the RTS phenotype to the chromosomal region between 14 and 22 Mb on BTA5. Our findings clearly demonstrate that the *RTS* and *dilution* loci are distinct loci on BTA5.

**Conclusions:**

Our study provides evidence that the *RTS* locus has effects on hair conformation and coat colour dilution and that the effect on coat colour dilution is clearly independent from that of the *dilution* locus. Finally, our results excluded several other loci that were previously reported to be associated with or to underlie hair conformation or pigmentation traits as the causal mutations of RTS and also several major functional candidate genes that are associated with hypotrichosis in humans. Our finding on the identification of a three-locus interaction that underlies RTS provides a prime example of epistatic interaction between several independent loci that is required for the expression of a distinct phenotype.

**Electronic supplementary material:**

The online version of this article (doi:10.1186/s12711-016-0199-8) contains supplementary material, which is available to authorized users.

## Background

**Fig. 1:**
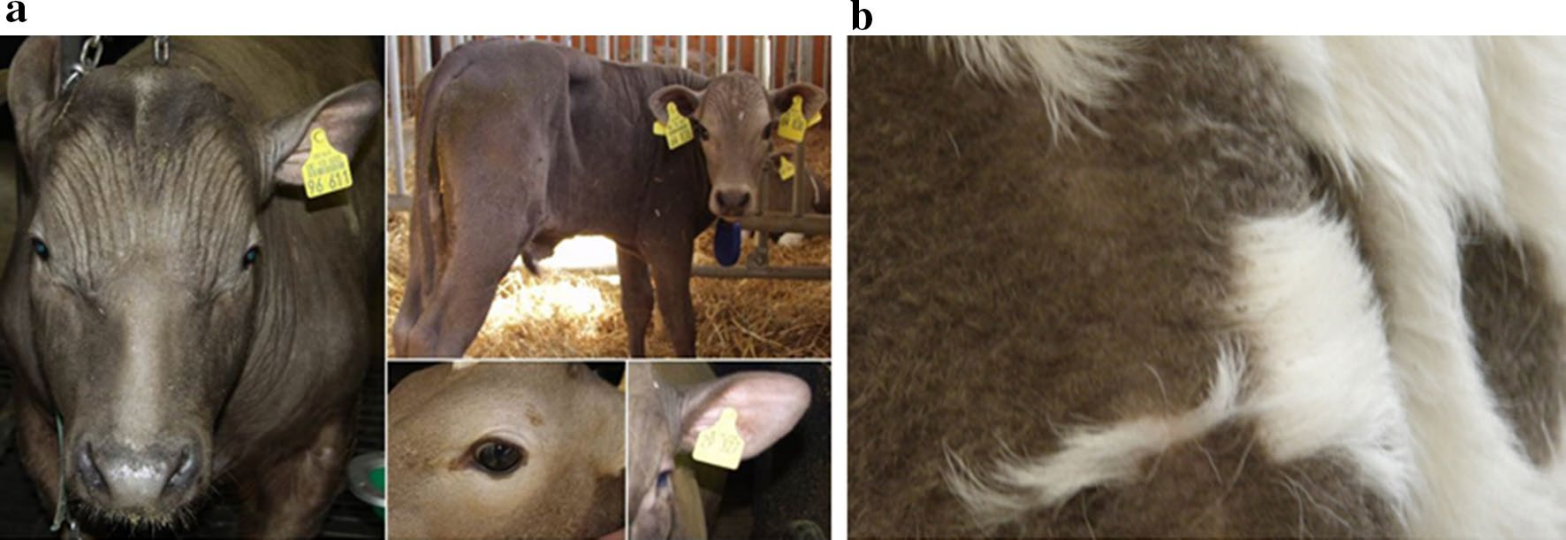
Phenotype of animals with the “rat-tail” syndrome (RTS) within the SEGFAM population. **a** Extreme cases of hypotrichosis, scarce hair, skin folds are visible; eye lashes and hair in the ears are absent; affected animals often lack normal tail switch development. **b** RTS individuals show variation in hair structure and length between pigmented and non-pigmented areas

Hypotrichosis is an inherited defect in mammals that is characterized by various degrees of sparse and curled malformed hair. In humans, a large number of causal mutations for hypotrichosis have been described [1]. The “rat-tail” syndrome (RTS) is a bovine congenital, inherited hypotrichosis that is characterized by misshaped, curly and sparse hair and by missing hairs at the tail switch, which gave this defect its descriptive name [2] (Fig. 1). The defect in hair conformation is restricted to the pigmented areas of hair coat, and the affected animals suffer from disturbed thermoregulation, which impairs their health and growth performance [3]. RTS occurs in crosses between black cattle breeds (e.g., Angus and Holstein) and some European breeds that are characterized by colour dilution of the coat (e.g., Simmental, Charolais and Hereford). Few reports found in the literature describe single cases of congenital hypotrichosis that is restricted to the pigmented skin areas in Black and White Holstein cows [4, 5]. The causal mechanism of this congenital malformation is unknown.

Schalles and Cundiff [3] postulated that RTS is caused by the epistatic interaction between two independent genetic loci. To date, affected cattle are assumed to carry an allele for black coat colour at one locus and to be heterozygous at a second locus. In Holstein and Angus cattle, the black coat colour is caused by the black *E*^D^ allele at the *extension* locus that corresponds to the *melanocortin 1 receptor* (*MC1R*) gene on *Bos taurus* autosome (BTA) 18; allele *E*^D^ is dominant over the *E*+ wild-type allele and the recessive red *e* allele [6, 7]. There are several reports in the literature that indicate that mutations in the *premelanosome protein* (*PMEL*) gene [also known as *silver* gene (*SILV*) on BTA5] are responsible for the *dilution* locus and that this gene corresponds to the second locus involved in RTS [8, 9]. However, in previous studies, we showed that neither mutations in the coding and regulatory regions of the *PMEL* gene, nor splicing variants of this gene are associated with RTS [10, 11]. Moreover, there are several reports on mutations that affect hair length and/or structure in Belted Galloway, Hereford and Fleckvieh cattle. Marron and Beever [12] and Markey [13] postulated that mutations in the *hephaestin*-*like 1* (*HEPHL1*) gene on BTA29 and the *keratin 71* (*KRT71*) gene on BTA5 are causal for variations in hair structure (HS) that are similar to RTS in Belted Galloway and Hereford, respectively. In addition, a recent study on the Fleckvieh breed reported a causal mutation in the *keratin 27* (*KRT27*) gene on BTA19 for curly hair [14], which is a phenotype that has also been associated with RTS. However, it should be noted that these mutations affect both pigmented and non-pigmented hair, have no effect on hair length, and/or are present on a pheomelanic phenotypic background. These characteristics are clearly distinct from those of RTS. Thus, because of these partly controversial results, the complete genetic mechanism that underlies RTS is still under debate and our aim was to monitor the genetic architecture of RTS and to map one (or several) locus(i) that are involved in epistatic interaction in a German Holstein × Charolais F_2_ cross population (i.e. SEGFAM population [15]), segregating for RTS.

## Methods

### Animals, traits and sampling

All experimental procedures were carried out according to the German animal care guidelines and were approved and supervised by the relevant authorities (Landesamt für Landwirtschaft, Lebensmittelsicherheit und Fischerei Mecklenburg-Vorpommern, Rostock; Landkreis Bad Doberan, Bad Doberan, Germany) of the State Mecklenburg-Vorpommern, Germany.

This study included individuals from the experimental resource cross population (SEGFAM) that was started in the P_0_ generation by crossing purebred German Holstein cows with purebred Charolais sires at the Leibniz Institute for Farm Animal Biology (FBN) [15]. This population was established by multiple ovulation and embryo transfer, which resulted in large half-sib and full-sib families. By crossing the dams from generation F_2_ with German Holstein bulls, two large paternal half-sib backcross (BC) families were generated. For our analyses, we used 740 individuals from the P_0_, F_1_, F_2_ and BC generations. From the BC generation, only the calves with grey hair (genotype combination *E*^D^/*, *D*c/*d*c+ at the *extension* (*MC1R*) [6] and *dilution* (*PMELc.64G*>*A*) loci, respectively [10]). For the different mapping designs, we analysed subsets of the full pedigree.

Since expression of the RTS phenotype varies among animals, for a precise dissection of the phenotype, all the animals were scored for hair conformation and pigmentation traits that are associated with RTS by double visual examination at 1 to 2 months of age (for the F_1_, F_2_, and BC generations) and after puberty (at more than 15 months of age for the F_1_ and F_2_ generations) by two independent persons. The recorded traits were: degree of colour dilution of the coat in pigmented areas (hereafter termed Dilu for dilution), difference in hair length between pigmented and non-pigmented body areas (HLV for hair length variation), HS and hair density (HD) as well as an overall classification score (RT) taking into account all hair conformation traits associated with RTS. Differential scoring of RTS-associated traits was performed as described in Additional file 1: Figure S1.

For DNA genotyping, leukocytes were purified from blood samples and stored at −20 °C until isolation of genomic DNA by phenol–chloroform standard methods. For the P_0_ and BC German Holstein sires, genomic DNA was extracted from sperm cells.

### Genotyping coat colour loci

Genotypes at the *extension* locus were determined by sequencing a region of the corresponding *MC1R* gene. First, a bovine-specific 444-bp *MC1R* sequence was amplified from genomic DNA by PCR using the following *MC1R* primers: MC1R F2: 5′-CCAGCCACCCTCCCCTTCACC-3′ and MC1R R2: 5′-CGCAATGATCCTCCACGCTCG-3′ that flank the causal mutations of alleles *E*^D^ (dominant black, eumelanin), *E*+ (wild type) and *e* (recessive red, pheomelanin). PCR was performed as follows: an initial denaturation step at 95 °C for 2 min, 12 cycles in a 2 °C-touchdown protocol starting at an annealing temperature of 68 °C (30 s 95 °C, 1 min 68 °C, 30 s 72 °C), 29 cycles of amplification at 55 °C (30 s 95 °C, 1 min 55 °C, 30 s 72 °C) and finally an elongation step at 72 °C for 7 min using the GoTaq^®^ G2 Hot Start polymerase (Promega). Then, the PCR products were sequenced using the primer MC1R F1: 5′-TACTACTTTATCTGCTGCCTG-3′ on a capillary sequencer (ABI 310, Applied Biosystems; MEGABACE, GE Healthcare) with BigDye^®^ (Applied Biosystems) chemistry. Alleles were identified as described by Klungland et al. [6].

To identify the genotypes at the *dilution* locus [10], a region of the bovine *PMEL* gene was amplified from genomic DNA, then digested with the restriction enzyme *BfmI* (Fermentas), and alleles *D*c (dominant diluted) and *d*c+ (recessive undiluted, wild type), which correspond to the *PMELc.64A* and *c.64G* alleles were analysed as described in [10].

### Segregation analysis

Segregation analysis of the RTS-associated traits was performed for 598 SEGFAM individuals from the F_2_ and BC generations and for which scores for the five analyzed traits i.e. HS, HD, HLV, Dilu and RT, and genotypes at the *extension* and *dilution* loci were available. Four individuals were excluded from the final analysis because they carried an *E*+ allele at the *extension* locus, which is present at a low incidence within the population used.

### Whole-genome SNP genotyping

Among the 740 SEGFAM animals, we genotyped all P_0_ sires, F_1_ and F_2_ individuals, and all grey BC calves (*E*^D^/*, *D*c/*d*c+) and their German Holstein sires with the BovSNP50 v1/v2 Bead Chip (50k) or the BovLDv1 Bead Chip (6k) according to the manufacturer’s instructions (Illumina Inc.,). After quality control using the Illumina Genome Studio v2011 (Illumina Inc.,) software and setting the following criteria: a call rate of at least 90 % for the 50k and 95 % for the 6k single nucleotide polymorphism (SNP) panels, a call frequency of at least 97 %, a GenTrain score of 0.60 and a minor allele frequency higher than 0.01, we retained 6802 (6k) and 37,218 (50k) SNPs for the linkage and association analyses, respectively. All genotypes were checked for Mendelian inconsistencies by using the PedCheck v1.1 program with levels 1 and 2 [16] and the PLINK v1.07 for IBD estimation [17].

### Linkage and association analysis

First, we obtained a preliminary genomic localization of the *RTS* locus by genome-wide quantitative trait loci (QTL) analyses as described by Hanna et al. [18]. We fitted additive and additive-dominant linear regression models within the GRIDQTL software package [19] to identify regions that affect hair malformation i.e. for the scores for HS, HD, HLV, and RT. First, only sex was included as fixed effect to account for known differences in hair pigmentation between males and females. Subsequently, we also included the genotypes at the *extension* and *dilution* loci as fixed effects to account for potential interactions between the *RTS* locus and these two loci. Linkage analyses were conducted on the 6k genotyping data in 1 cM steps based on the physical positions of SNPs according to the UMD3.1 assembly of the *B. taurus* genome [20] and the simplistic assumption that 1 cM ~1 Mb as in [18]. Chromosome- and genome-wide significance thresholds were determined by data permutations with 10,000 replicates and 95 % confidence intervals were obtained based on 10,000 bootstrap samples. For the analyses using the F_2_ full-sib families, we included the genotypes for generations P_0_, F_1_ and F_2_ and the phenotypes for generation F_2_. For the linkage analyses using the BC half-sib families, we included the genotypes for generations F_2_ and BC and for the German Holstein sires that were used for backcrossing and the phenotypes for the BC generation.

To verify our preliminary results, we conducted additional non-parametric linkage analyses using the MERLIN v1.1.2 software package (multipoint engine for rapid likelihood inference, [21]), based on the multipoint algorithm according to [22]. To this end, we selected the individuals with a eumelaneic dilute-coloured coat (*E*^D^/*, *Dc*/*dc*+) and rescored the animal phenotypes as “unaffected” (score 1), “affected” (scores 3 and 4) and “missing” (score 2, when the status was ambiguous) for the most discriminating RTS-associated trait, HLV. In our analyses, we used the exponential model of MERLIN [23] to test for linkage.

Finally, we performed a genome-wide association study (GWAS) using the SNP and Variation Suite v8 (Golden Helix, Inc., www.goldenhelix.com) to refine the localization of the *RTS* locus. For the GWAS, we selected the individuals from generations F_1_, F_2_ and BC that fulfilled the precondition for RTS as determined by the segregation analysis, i.e. animals with at least one *E*^D^ allele at the *extension* locus and that were heterozygous for the *PMEL* c.64G>A locus. We calculated a genomic relationship matrix, which was included in a single locus mixed linear model (EMMAX, [24]) to account for relatedness and stratification effects within the dataset. Input variables were the scores for RT, HLV, HS, HD and Dilu. In addition to the linear mixed model, we also performed a case–control study, which required recoding of scores for RT, HLV and Dilu. For RT and HLV, all individuals were recoded essentially as described for the non-parametric linkage analyses. For the Dilu trait, since we had already excluded all individuals with a phenotype scored as 1, we coded all animals with a score 2 as “affected”, all animals with a score 4 as “unaffected” and all animals with a score 3 as “missing” for the case–control design. These recoded phenotypes were used for a case–control association study assuming an additive mode of inheritance and including 10 principal components to account for stratification effects. To account for multiple-testing in the linear mixed model as in the case–control study, we applied a Bonferroni correction of nominal p values.

### Transcriptome analysis

Skin samples were collected after slaughter from two piebald F_2_ bulls (one with RTS phenotype and one individual with wild type phenotype) from adjacent pigmented and non-pigmented areas of the neck essentially as described previously in [25]. In addition, we included pigmented skin samples from two other F_2_ bulls with RTS phenotype to confirm the initial results. For each sample, the skin was trimmed from fat tissue and snap-frozen at −80 °C. RNA and library preparation for RNAseq using the Illumina Truseq RNA sample prep kit (Illumina Inc.,) was carried out as described in [25]. After quality control, the respective libraries were sequenced with a 2 × 61 bp paired-end protocol on an Illumina GAIIx sequencer. Quality control and alignment of reads including subsequent bioinformatic analyses were conducted as described in [25]. The resulting raw BAM files from the initial alignment of reads were visually screened for differences in the exon–intron structure of the genes that are located in the region between 10 and 25 Mb on BTA5 and are known to be transcribed in the RTS and/or control animals. The gff3 file for the *B. taurus* annotation Release 104 of the bovine genome [26] and.gtf file [25] for project-specific gene annotation served as references for the genes that were located within the target region.

## Results

### Segregation analysis and hypothesis

The dataset used in this study included 50 P_0_, 69 F_1_, 409 F_2_ and 212 BC individuals from the SEGFAM cross population. A total of 598 (393 F_2_ and 205 BC) individuals with scores for each of the hair pigmentation and conformation traits were selected and used for the segregation analysis, which showed that a distinct RTS phenotype (RT scores of 3 and 4, Table 1) was restricted to individuals with the genotype combination (*E*^D^/*, *D*c/*d*c+). However, among all 176 F_2_ and 205 BC animals with this genotype combination, about 50 % displayed a RTS phenotype ((94 and 124 with an RT score of 2 to 4 for the F_2_ and BC animals, respectively) and the remaining individuals displayed no indication on RTS phenotype. Two F_2_ individuals had an RT score of 2 but were genotyped *E*^D^/*, *D*c/*D*c. No individual with a pheomelanic coat colour background (*e*/*e* at the *extension* locus) or with a “crème” or undiluted coat colour (homozygous for the mutant or wild type allele at the *PMEL c.64G*>*A* locus) showed an RTS-associated hair conformation.

**Table 1:** Segregation analysis of 598 F_2_ and backcross (BC) SEGFAM calves based on the RTS classification score (RT)

Coat colour	*MC1R*	*PMEL*	F_2_			BC		
			Non-affected RT 1	RT 3–4	RT 2	Non-affected RT 1	RT 3–4	RT 2
Black, no dilution	*E* ^D^/*	*d*c+/*d*c+	40	0	0	0	0	0
Black, moderate dilution	*E* ^D^/*	*D*c/*d*c+	85	36	58	81	48	76
Black, strong dilution	*E* ^D^/*	*D*c/*D*c	75	0	2	0	0	0
Red, no dilution	*e*/*e*	*d*c+/*d*c+	21	0	0	0	0	0
Red, moderate dilution	*e*/*e*	*D*c/*d*c+	57	0	0	0	0	0
Red, strong dilution	*e*/*e*	*D*c/*D*c	22	0	0	0	0	0

* Either allele *E* ^D^ (dominant allele for black basic coat colour) or *e* (recessive, red allele) at the *extension* (*MC1R*) locus; *d*c+ recessive wild type allele (no coat colour dilution) and *D*c dominant allele (coat colour dilution) at the *dilution* (*PMEL c.64G*>*A*) locus

### Mapping the RTS locus by linkage analysis

**Fig. 2:**
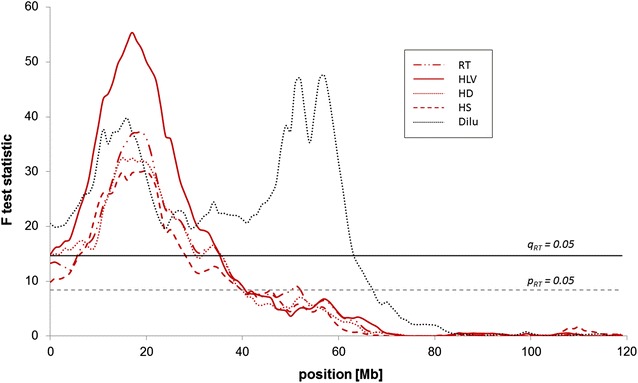
F test statistic of the linkage analysis on BTA5 within the F_2_ design. *Dilu* level of coat colour dilution in pigmented areas, *HLV* level of hair length variation between pigmented and non-pigmented coat, *HS* hair structure, *HD* hair density, *RT* RTS classification score phenotype

Initially, we obtained genome-wide significant signals on BTA5 for all hair conformation and pigmentation traits by using an additive-dominant model across all autosomes and the pseudoautosomal region of the sex chromosomes and by fitting sex as fixed effect within an F_2_ design that comprised 388 F_2_ individuals. Including genotypes from the 6k SNP panel consistently identified SNPs with high information content (> 0.95) across the whole genome (data not shown). The highest peaks of the test statistic were coincident with the position of the *PMEL* gene between 55 and 57 Mb on BTA5 (see Additional file 2: Figure S2). However, the test statistic also showed a second peak exceeding the genome-wide significance threshold at 18 Mb. Based on the results from the segregation analysis, which had indicated that, in addition to the *extension* and *dilution* loci, a third locus was involved in the mechanism underlying RTS, we subsequently performed a genome-wide linkage analysis by fitting an additive model with sex and the genotypes for *MC1R* and *PMELc.64G>A* as fixed effects. This analysis revealed a single locus with genome-wide (q < 0.01) significant signals on BTA5, which affected all hair conformation traits (HS, HD, HLV, RT) (Fig. 2; Table 2). For all hair conformation traits, a single peak between 18 and 20 Mb on BTA5 was observed, whereas two distinct peaks exceeding the genome-wide significance threshold were detected for Dilu at 18 and 57 Mb, respectively. A 2-QTL model confirmed that in addition to the *PMEL c.64G*>*A* locus, there were two segregating loci that were located on BTA5 and affected hair pigmentation dilution (F value for the 2-QTL model versus 1-QTL model: 37.46 (see Additional file 3: Figure S3). In addition to the genome-wide significant signal on BTA5, we detected chromosome-wide (p < 0.05) significant signals using the F_2_ design for HLV and HS on BTA6 and 23, respectively, and for HS on BTA26 (Table 2).

**Table 2:** Results of the linkage analysis for RTS-associated hair pigmentation and pigmentation traits within the F_2_ design

BTA	Trait	Position^a^	F value	p value	q value	CI (Mb)	QTL effect	
							Est. effect	SE
5	Hair structure (HS)	20	30.10	<0.0001	<0.0001	10.0–22.0	−0.48	0.09
5	Hair density (HD)	15	32.51	<0.0001	<0.0001	7.0–27.0	−0.29	0.05
5	Hair lengths variation (HLV)	17	55.37	<0.0001	<0.0001	11.0–22.0	−0.42	0.06
5	Degree of coat colour dilution (Dilu)	57	47.60	<0.0001	<0.0001	11.0–57.0	0.94	0.14
5	Degree of coat colour dilution (Dilu)	16	39.75	<0.0001	<0.0001			
5	RTS classification phenotype (RT)	19	37.21	<0.0001	<0.0001	14.0–22.0	−0.31	0.05
6	Hair lengths variation (HLV)	71	10.32	0.0206	ns	21.0–112.0	−0.16	0.05
23	Hair structure (HS)	34	10.22	0.0123	ns	14.0–47.0	−0.24	0.07
26	Hair structure (HS)	20	8.20	0.0307	ns	0.0–43.0	−0.22	0.08

^a^Position in Mb; p value: chromosome-wide significance, q value: genome-wide significance, ns: not significant, CI: 95 % confidence interval in Mb, Est. effect: estimated locus effects and the respective standard error (SE)

**Fig. 3:**
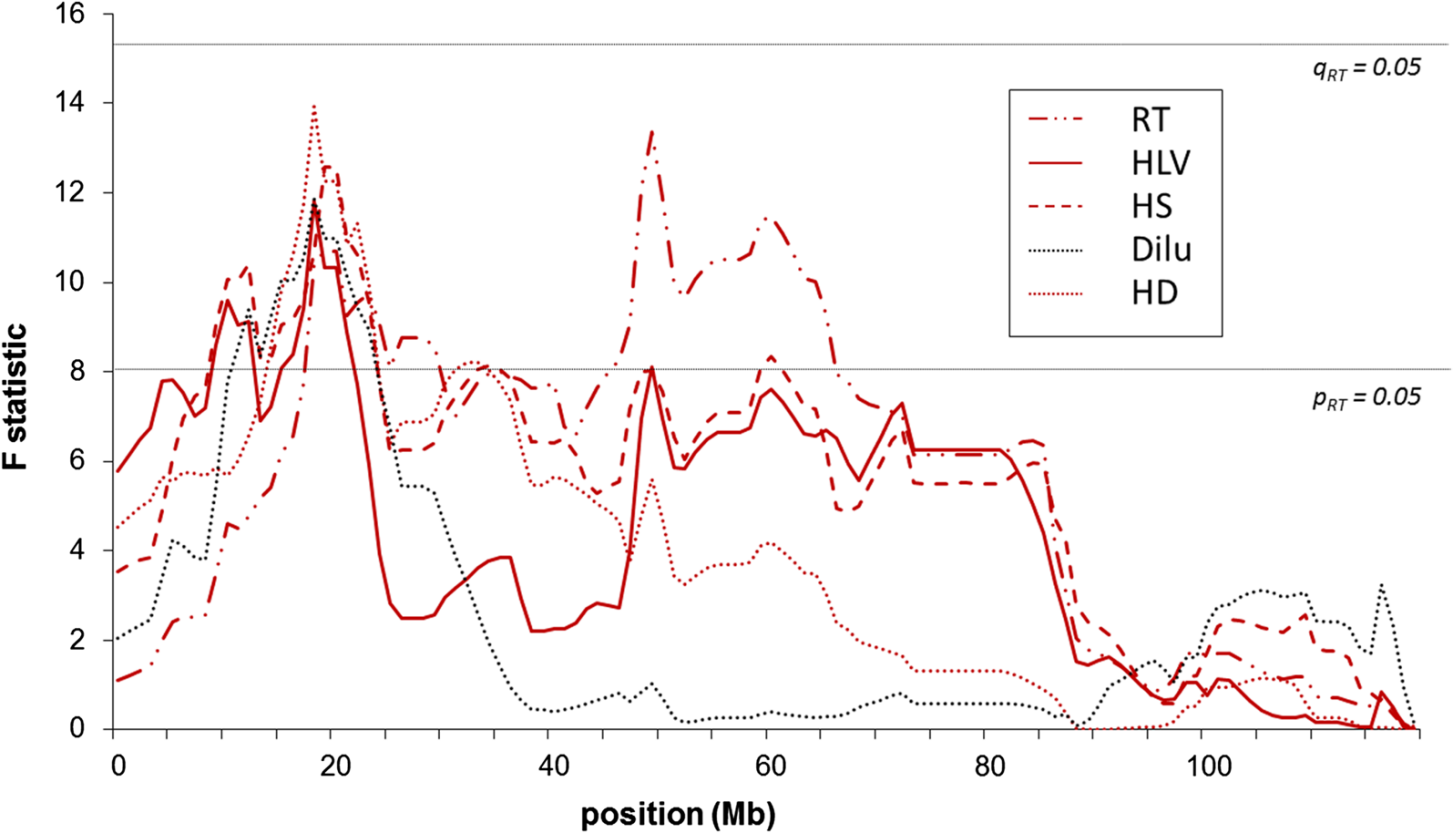
Test statistic of the linkage analysis on BTA5 in the half-sib sire 1 family. *Dilu* level of coat colour dilution in pigmented areas, *HLV* level of hair length variation between pigmented and non-pigmented coat, *HS* hair structure, *HD* hair density, *RT* RTS classification score phenotype

The availability of two large BC half-sib groups that were generated from two German Holstein sires (97 and 109 BC calves from sires 1 and 2, respectively) allowed us to perform independent half-sib linkage analyses that tested the German Holstein sires for segregation of loci that affect RTS-associated hair conformation and pigmentation traits. Table 3 shows that the two sires did not share a chromosomal region that segregated for RTS-associated traits. A chromosome-wide significant locus that affects all hair conformation traits was detected on BTA5 between 18 and 20 Mb with the sire 1 family and confirmed the initial results obtained with the F_2_ design (Fig. 3). Interestingly, as with the F_2_ design, two peaks were detected for the RT trait. It should be noted that sire 1 had an undiluted phenotype that is characteristic of Holstein black and white cattle. With the sire 1 family, a genome-wide significant locus for hair conformation traits (particularly HS) was detected on BTA10, and several chromosome-wide significant loci on BTA9, 19, 25, 26 and 29, in addition to the locus on BTA5 for RTS-associated traits. With the sire 2 family, a genome-wide significant locus for RT on BTA20 (see Additional file 4: Figure S4) and several chromosome-wide significant loci for one or more RTS-associated traits on BTA3, 9, 13, 14, 18, and 23 were detected. On BTA18, the *extension* locus was more than 30 Mb away from the region that segregated for Dilu and HLV and outside the confidence interval for Dilu. Concordant genomic mapping of several loci for different hair conformation traits at the same position suggested that some genes that impact variation in hair traits may have pleiotropic effects.

**Table 3:** Results of the linkage analysis for RTS-associated hair pigmentation and pigmentation traits within a half-sib backcross (BC) design for each of the two sire sib ships

BTA	Trait	Position^a^	F value	p value	q value	CI (Mb)	Sire	Locus effect	
								Est. effect	SE
3	Hair length variation (HLV)	83	9.35	0.0303	ns	13.0–113.0	2	−0.63	0.20
5	Hair structure (HS)	20	10.93	0.0130	ns	8.0–105.5	1	−1.01	0.31
5	Hair density (HD)	18	14.80	0.0033	ns	5.0–63.0	1	−0.66	0.18
5	Hair length variation (HLV)	18	11.42	0.0126	ns	4.0–80.0	1	−0.70	0.21
5	Level of coat colour dilution (Dilu)	18	11.17	0.0116	ns	10.0–116.0	1	0.38	0.12
5	RTS classification phenotype (RT)	49	13.16	0.0072	ns	15.0–65.0	1	−0.67	0.18
8	Hair structure (HS)	44	10.49	0.0210	ns	8.0–107.0	1	−0.99	0.30
8	Hair length variation (HLV)	38	10.25	0.0201	ns	4.0–101.0	1	−0.69	0.21
8	RTS classification phenotype (RT)	13	9.01	0.0366	ns	8.0–94.0	1	−0.56	0.19
9	Hair structure (HS)	90	9.20	0.0205	ns	57.0–98.0	2	0.74	0.25
9	Hair density (HD)	98	7.97	0.0371	ns	50.0–103.0	2	0.56	0.20
9	Hair length variation (HLV)	90	10.24	0.0147	ns	35.0–96.0	2	0.64	0.20
9	RTS classification phenotype (RT)	98	8.42	0.0326	ns	41.0–101.0	2	0.50	0.17
10	Hair structure (HS)	96	18.08	0.0011	0.0207	10.0–98.0	1	1.24	0.29
10	Hair density (HD)	96	12.48	0.0076	ns	39.0–103.0	1	0.60	0.17
10	RTS classification phenotype (RT)	94	10.51	0.0160	ns	0.0–103.0	1	0.61	0.19
13	Hair density (HD)	58	14.53	0.0026	ns	23.0–62.0	2	0.73	0.19
13	RTS classification phenotype (RT)	42	12.47	0.0067	ns	23.0–70.0	2	0.59	0.17
14	RTS classification phenotype (RT)	36	9.80	0.0170	ns	13.0–75.0	2	−0.53	0.17
18	Hair structure (HS)	46	10.72	0.0104	ns	0.0–51.0	2	0.80	0.25
18	Hair length variation (HLV)	43	14.55	0.0030	ns	12.0–48.0	2	0.76	0.20
18	Level of coat colour dilution (Dilu)	46	15.19	0.0020	ns	40.0–56.0	2	−0.39	0.10
19	Hair structure (HS)	8	9.42	0.0250	ns	7.0–53.0	1	0.95	0.31
20	Hair density (HD)	70	14.05	0.0025	ns	44.0–70.0	2	−0.71	0.19
20	RTS classification phenotype (RT)	57	19.33	0.0003	0.009	46.5–70.0	2	−0.71	0.16
23	RTS classification phenotype (RT)	6	9.07	0.0192	ns	3.0–51.0	2	0.51	0.17
25	Hair structure (HS)	5	10.25	0.0127	ns	0.0–36.0	1	0.98	0.31
25	Hair density (HD)	5	8.29	0.0265	ns	2.0–39.0	1	0.51	0.18
25	Hair length variation (HLV)	5	10.19	0.0108	ns	1.0–36.0	1	0.67	0.21
25	RTS classification phenotype (RT)	4	9.00	0.0194	ns	0.0–40.0	1	0.57	0.19
26	Hair density (HD)	36	9.01	0.0220	ns	0.0–48.0	1	−0.53	0.18
29	Hair density (HD)	47	8.71	0.0226	ns	0.0–49.0	1	−0.52	0.18
29	RTS classification phenotype (RT)	47	10.51	0.0124	ns	3.0–49.0	1	−0.60	0.19

^a^Position in Mb; p value: chromosome-wide significance, q value: genome-wide significance, ns: not significant, Sire: sire family significantly segregating for the respective locus, CI: 95 % confidence interval in Mb, Est. effect: estimated locus effects and the respective standard error (SE)

Additional non-parametric analyses using MERLIN were performed using F_1_ and F_2_ individuals and showed significant signals on BTA5 at 15 Mb for the most discriminating hair conformation trait HLV (q < 0.01, after accounting for testing 30 chromosomes) and confirmed the initial results from the linear regression QTL analysis.

### Genome-wide association study

**Fig. 4:**
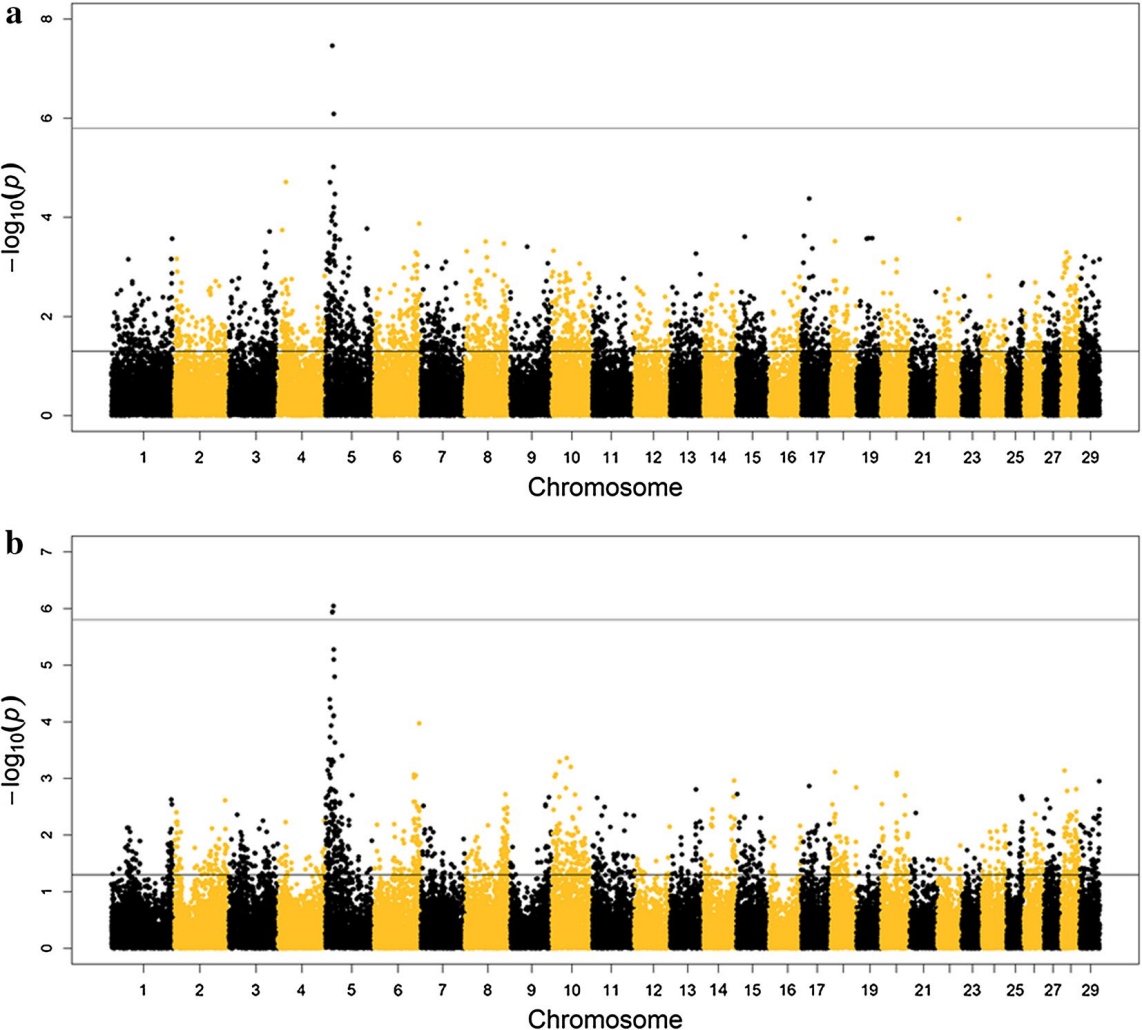
Manhattan plot of the whole-genome association analysis for RTS classification score (RT) phenotype. **a** Fitting a single-locus mixed model (MM) including a genomic relationship matrix. **b** Case–control study (CC) for the trait RTS classification score (RT) phenotype. The *upper horizontal line* represents the genome-wide significance threshold q = 0.05, the *lower horizontal line* represents the nominal p value = 0.05

We performed a GWAS to further narrow down the localization of the locus/loci that underlie RTS. When only grey (*E*^D^/*, *D*c/*d*c+) F_1_, F_2_ and BC individuals were included, genome-wide significant signals were detected on BTA5 with the linear mixed model as well as with the case–control design, which confirmed the results of the initial linkage analyses (Fig. 4; Additional file 5: Figure S5). With the mixed linear model and using the non-recoded phenotype scores, we obtained genome-wide significant associations for HLV and RT (Table 4). SNP BTA-74302-no-rs on BTA5 (located at 18,047,140 bp) was the most significant SNP for both traits. In the case–control design, we obtained genome-wide significant results for HLV, RT and Dilu, and SNP BTA-74302-no-rs was again highly significant with the lowest q value of all SNPs for HLV and was among the three markers with the most significant associations for RT and Dilu in our GWAS. This SNP had contrasted allele frequencies in the Holstein and Charolais P_0_ founder animals i.e. frequency of allele 1_Charolais_ = 0.90 and frequency of allele 1_Holstein_ = 0.32). In spite of its significant association signals, this SNP was not fully associated with RTS (see Additional file 6: Table S1). The SNP that showed the most significant association with RT and Dilu was Hapmap44614-BTA-72802 at position 21,114,508 bp on BTA5. For all trait-model combinations, except the mixed linear model for HLV, the three SNPs with the strongest genome-wide significance were located in the interval between 18.04 and 21.11 Mb. This position is within the confidence intervals for the *RTS* locus that were determined by linkage analysis with the F_2_ and the BC subsets.

**Table 4:** Summary of genome-wide significant associations from a GWAS for hair conformation and hair dilution traits associated with the RTS in F_1_, F_2_ and BC individuals with at least one *E* ^D^ allele at the *extension* locus and heterozygous for the *PMEL* *c.64G*>*A* locus

Trait	Model^a^	Marker	BTA	Position (bp)	p value	q value^b^
Level of coat colour dilution	Case–control	Hapmap44614-BTA-72802	5	21,114,508	8.90E−07	0.033
Level of coat colour dilution	Case–control	Hapmap53597-rs29010787	5	19,015,523	1.15E−06	0.043
Level of coat colour dilution	Case–control	BTA-74302-no-rs	5	18,047,140	1.22E−06	0.046
Hair length variation	Case–control	BTA-74302-no-rs	5	18,047,140	1.74E−07	0.007
Hair length variation	Case–control	Hapmap42402-BTA-103350	5	21,484,201	2.69E−07	0.010
Hair length variation	Case–control	Hapmap44614-BTA-72802	5	21,114,508	1.04E−06	0.039
Hair length variation	Case–control	ARS-BFGL-NGS-26310	5	12,123,561	1.17E−06	0.044
RTS classification phenotype	Case–control	Hapmap44614-BTA-72802	5	21,114,508	9.03E−07	0.033
RTS classification phenotype	Case–control	Hapmap53597-rs29010787	5	19,015,523	1.14E−06	0.043
RTS classification phenotype	Case–control	BTA-74302-no-rs	5	18,047,140	1.17E−06	0.044
Hair length variation	Mixed	BTA-74302-no-rs	5	18,047,140	7.36E−09	0.0003
Hair length variation	Mixed	ARS-BFGL-NGS-26310	5	12,123,561	1.68E−07	0.006
Hair length variation	Mixed	BTA-103542-no-rs	5	16,795,347	1.84E−07	0.007
RTS classification phenotype	Mixed	BTA-74302-no-rs	5	18,047,140	3.43E−08	0.001
RTS classification phenotype	Mixed	Hapmap42402-BTA-103350	5	21,484,201	8.16E−07	0.030

^a^Case–control: genotype association study in a case–control design, Mixed: mixed linear models including phenotype scores

^b^Bonferroni corrected p values to account for multiple testing

After the effects of the *extension* and *dilution* loci were fitted, including a dominant effect in the model did not increase the test statistic compared to that of an additive effect in the linkage or the association analyses for RT, HLV, HD or KRT. The only genome-wide significant association (q = 0.005) that was detected in the dominant mixed model association analysis was between Dilu and SNP Hapmap27767-BTA-154179 located at 60,263,049 bp on BTA5.

### Transcriptome analysis

In the region between 10 and 25 Mb on BTA5, 102 genes are annotated in Release 104 of the *B. taurus* genome assembly. For all the samples investigated by RNAseq, more than 35 million uniquely mapped fragments were available for whole-transcriptome data analysis. Genes with at least ten aligned reads were assigned the status “expressed”. From the 102 genes, 37 exceeded this threshold (see Additional file 7: Table S2), but none were expressed exclusively in the skin of animals with RTS or in wild type pigmented or unpigmented skin. In addition, although we observed alternative splicing for several genes, no RTS-associated alternative transcript isoforms were observed for any of the genes between RTS and wild type pigmented skin (see Additional file 7: Table S2). Transcripts for the *POC1 centriolar protein B* (*POC1B*) and *FYVE*, *RhoGEF and PH domain containing 6* (*FGD6*) genes were detected in one pigmented RTS skin that differed from wild type skin, but this was not consistent across all three pigmented RTS samples.

## Discussion

Segregation of RTS is present within the experimental cross SEGFAM [15] population between black German Holstein and Charolais breeds. At the *extension* locus, black German Holstein cattle carry at least one dominant black allele (*E*^D^), whereas Charolais are homozygous (*e*/*e*) for the recessive allele. However, due to homozygosity of the mutant *D*c allele at the *dilution* locus, Charolais cattle express an extremely diluted coat colour (“crème”) instead of the red undiluted pigmentation [10]. The homozygous recessive wild type genotype (*d*c+/*d*c+) that is present in German Holstein cattle causes an undiluted basic coat colour. By crossing these two breeds, offspring with the genotype combination *E*^D^/*, *D*c/*d*c+ are generated, which agrees with the initial hypothesis of a two-locus model i.e. the *extension* and *dilution* loci as the causal mechanism for RTS phenotypes described by Schalles and Cundiff [3]. However, our results from the segregation analysis prove that this two-locus model is not sufficient to fully explain the RTS phenotype. Although RTS obviously requires the expression of a diluted eumelanic pigmentation background (Table 1) that fits with the initial hypothesis of Schalles and Cundiff [3], our data provide evidence that epistatic interaction with at least a third independent locus is required for the expression of the RTS phenotype. This finding contrasts with reports about causal mutations for RTS within the *PMEL* locus [8, 9]. Our data based on linkage and association analyses also support the conclusions from the segregation analysis. Both linkage analysis with the full-sib F_2_ and half-sib BC designs and association analysis with a population comprising F_1_, F_2_ and BC animals, mapped RTS-associated hair conformation traits between 15 and 20 Mb on BTA5, which is far from the *PMEL* gene at 57.6 Mb. *PMEL* is outside the confidence interval for RTS-associated traits as determined by linkage analysis bootstrapping. Correspondingly, the genotype combination of *E*^D^/*, *D*c/*d*c+ is required, but not sufficient for the expression of the RTS phenotype. Thus, we postulate the existence of a third locus that plays a role in the RTS phenotype and is in epistatic interaction with the *extension* and *dilution* loci. Otherwise all or almost all grey individuals with the genotype combination *E*^D^/*, *D*c/*d*c+ at the *extension* and *dilution* loci would display the RTS phenotype. An autosomal additive model of inheritance for RTS is suggested, because after fitting the effects of the *extension* and *dilution* loci, including a dominant effect did not increase the test statistic in the linkage or association analyses for RTS-associated traits except for Dilu.

In dogs, black hair follicular dysplasia (BHFD) is an inherited recessive defect that also specifically affects pigmented hair in piebald individuals [27]. BHFD has a recessive mode of inheritance and is characterized by coat colour dilution, hypotrichosis and hair conformation defects. BHFD is assumed to be identical to the coat colour dilution (CDA) defect observed in some non-piebald breeds with dilute coat colour (e.g., Pinschers). Interestingly, Welle et al. [28] also postulated that, in addition to a gene causing coat colour dilution, another factor was required for the expression of the CDS/BHFD phenotype. While for several dog breeds with BHFD/CDA, *melanophilin* (*MLPH*) was identified as the causative gene for coat colour dilution [29], in cattle, mutations in the *PMEL* gene are causal for coat colour dilution and represent one epistatic factor for RTS.

By using several designs (full sib F_2_, half-sib BC, joint F_1_, F_2_, and BC populations) and statistical analyses (linkage and association mapping), we were able to show that the third epistatic locus involved in RTS (in addition to the *extension* and *dilution* loci) mapped to BTA5 between 14 and 22 Mb. This genuine *RTS* locus obviously affects HS as well as coat colour dilution (Table 2). The hypothesis of a third locus is supported by the results of the GWAS, which included only animals with a heterozygous genotype at the *PMEL c.64G*>*A* locus and showed a significant association between Dilu and SNP BTA-74302-no-rs. This association signal was located exactly at the same position as the locus for RTS-associated hair conformation traits. Further evidence for more than one locus affecting coat colour dilution was provided, because fitting the *PMEL c.64G*>*A* genotype as a fixed effect into the linkage analysis did not fully eliminate a highly significant peak in the test statistic on BTA5 for coat colour dilution in the F_2_ design, which had been obtained in a model without this fixed effect. Instead, two individual peaks at 16 and 57 Mb were observed in the extended model with the *PMEL c.64G*>*A* genotype as a fixed effect. This indicates that the *RTS* locus located between 14 and 22 Mb also affects coat colour dilution and that the chromosomal region that includes the *PMEL* gene contains information in addition to the *PMEL c.64.G*>*A* mutation that affects coat colour dilution.

When we first performed linkage analyses without fitting the genotypes at the *PMEL c.64G*>*A* and *extension* loci into the model, we obtained highly significant peaks for all monitored RTS-associated traits that were located on BTA5 between 55 and 57 Mb (see Additional file 2: Figure S2), which corresponds exactly to the localization of the *PMEL* gene. Thus, our data show that missing an essential factor in an epistatic interaction network will yield false positive results. We hypothesize that previous reports on the role of *PMEL* in the mechanism that underlies RTS [8, 9] may have suffered from this problem.

Our data from the linkage analyses on the F_2_ and BC populations also suggest that there are additional loci, which combined with the *extension* and *dilution* loci, cause hair conformation modifications. Genome-wide significant effects on HS and RTS classification score (RT) from loci on BTA10 and 20 that were detected by using single large half-sib families provide support for this hypothesis. To our knowledge, none of these loci have been detected in previous studies. However, we did not observe any significant linkage or association signal nearby the *HEPHL1* gene on BTA29 or the *KRT71* gene on BTA5, two genes which had been reported to harbour mutations responsible for variations in hair length or structure that share phenotypic similarities with RTS. These results suggest that there might be a substantial amount of genetic heterogeneity in phenotypes with “rat-tail”-like features. Furthermore, it demonstrates that a very detailed description of the hair phenotype is required to identify the respective causal mutations.

The keratin gene cluster is a very obvious functional candidate region located on BTA5. However, the position of this cluster is outside the confidence interval for RTS as determined by linkage analysis. In addition, no SNP located within the keratin cluster was associated with the overall “rat-tail” phenotype RT (p value <0.05). Thus, mutations within the keratin gene cluster are not likely causal mutations for RTS. Furthermore, all genes reviewed by Basit et al. [30] as causal for hypotrichosis in humans, were excluded as candidates for RTS based on positional data from our linkage and association studies: none of our models or designs indicated a significant locus in one of the respective orthologous bovine chromosomal regions.

Regarding potential candidate genes for RTS, the *KITLG* (*ligand for the receptor*-*type protein*-*tyrosine kinase KIT*) gene located at 18 Mb on BTA5 is a very obvious candidate based on its known role in pigmentation [31, 32]. However, sequencing the coding region and the proximal promoter region of the *KITLG* gene did not yield any variant associated with RTS (data not shown). This does not preclude that a regulatory region of the *KITLG* gene may contain a causal mutation for RTS, as demonstrated by Guenther et al. [33]. These authors showed that, in humans, a SNP that is located 350 kb upstream of the *KITLG* transcription start site, significantly alters the activity of a hair follicle associated enhancer and causes blond hair in North Europeans. Results from RNAseq-based holistic transcriptome analysis did not reveal any changes in the exon–intron structure of the currently annotated genes, which are located in the interval between 10 and 25 Mb on BTA5 and are expressed in the skin, between individuals with RTS and wild type cattle. However, we identified a number of exons in genes that have not yet been annotated but are expressed in pigmented and/or unpigmented skin. This demonstrates the need for further efforts to improve the functional genome annotation in cattle. Interestingly, Basit et al. [34] described a digenic mode of inheritance for hypotrichosis in two independent human families with one locus represented by mutations in the *cadherin 3* (*CDH3*) gene and a second locus located in a chromosomal region (HSA12q21.2-22) that is syntenic with a region on BTA5 around 18 Mb. As in our study, the authors did not identify any defect-associated mutation in the coding region or splice sites of six functional candidate genes including *KITLG* on HSA12q21.2-22.

## Conclusions

Our study provides evidence that the RTS phenotype results from an epistatic interaction between three independent loci: *dilution* (that corresponds to the *PMEL* gene at 55 Mb on BTA5), *extension* (that corresponds to the *MC1R* gene on BTA18) and the *RTS* locus that is located in the interval between 14 and 22 Mb on BTA5. The prerequisites for expression of the RTS phenotype are a eumelanic background due to the presence of the dominant *E*^D^ allele at *MC1R* (*extension* locus) and a heterozygous genotype at the *PMEL* gene variant *c.64G*>*A* (*dilution* locus). The positions of the *RTS* and *dilution* loci on BTA5 are clearly distinct. Finally, our results excluded several potential loci that were reported to be associated with RTS or that underlie hair conformation or pigmentation traits as the causal mutations of RTS and also several major functional candidate genes that are associated with hypotrichosis in humans. Additional studies are required to investigate whether RTS is caused by a mutation in a yet unknown functional element located in the target chromosomal region on BTA5 and to analyze the substantial genetic heterogeneity of the RTS phenotype. However, our finding on the identification of a three-locus interaction that underlies RTS provides a prime example of epistatic interaction between several independent loci that is necessary for the expression of a distinct phenotype.

## Additional files

10.1186/s12711-016-0199-8 Phenotype scoring of RTS associated hair conformation and pigmentation traits. Phenotypes and corresponding scores for HS: HS 1: wild type, straight hair, HS 2: hair with fluffy, smooth structure, HS 3: curly hair with a mildly frizzy/wiry structure, HS 4: coarse, frizzy/wiry hair, HS 5: very coarse/wiry hair, HS 6: extremely coarse/wiry HS. Phenotypes and corresponding scores for hair density (HD): HD 1: wild type, full, thick, long hair covering the whole body as well as in the ears and around the eyes, HD 2: scarce hair at the trunk and/or few, short hair in the ears and around the eyes, HD 3: short and sparse hair in the ears and around the eyes as well as thin coat, HD 4: without eye lashes and/or hair in the ears and with very thin hair coat and/or bald areas. Phenotypes and corresponding scores for hair length variations between pigmented and unpigmented areas of spotted animals (HLV): HLV 1: wild type, equally long hair in pigmented and unpigmented sections. HLV 2: pigmented hair mildly shorter than unpigmented hair, HLV 3: pigmented hair moderately shorter than unpigmented hair, HLV 4: pigmented hair extremely shorter than unpigmented hair. Animals without white spotting marks were scored according to their phenotype for RT. Phenotypes and corresponding scores for RTS classification phenotype: RT 1: wild type without any RTS-associated hair conformation malformation, RT 2: mild HLV and/or short hair in ears and short, thin eye lashes, reduced tail switch. RT 3: sparse, coarse hair, moderate HLV, few hairs in ears/around eyes and/or few hair in eye lashes, tail switch only rudimentary. RT 4: very sparse coat, extreme HLV, scarce/wiry hair in pigmented areas, no hair in ears, barely/no eyelashes, extremely visible skin folds at face/throat, bald areas surrounding the eyes/in the ears, missing tail switch. Status of tail switch was only informative, if the end of the tail was pigmented. Phenotypes and corresponding scores for coat colour dilution (Dilu) in pigmented coat sections on a eumelanic (black, brown) and pheomelanic (red, yellow) colour background. Dilu 1: undiluted coat colour, black or red-brown; Dilu 2: mild colour dilution with dark grey colour for the black/brown background and orange/caramel for the red background, Dilu 3: mouse grey/bright grey (black background) or yellow/beige (red background), Dilu 4: extremely diluted, with crème and nearly white pigmentation.
10.1186/s12711-016-0199-8 Test statistic for hair conformation and pigmentation traits on BTA5 from the F_2_ full sib design fitting sex as fixed effect in an additive-dominant model. HLV: level of hair length variation between pigmented and unpigmented body areas, HS: hair structure, HD: hair density, RT: RTS classification phenotype. The test statistic for Dilu is not shown due to excessively high F values (>500), which exceed the scale of the y axis.
10.1186/s12711-016-0199-8 Contour plot showing the test statistic for coat colour dilution (Dilu) on BTA5 for a 2-QTL model in a F_2_ full sib design. The x and y axes represent chromosomal positions for QTL1 and QTL2, respectively. The z axis indicates the F value of the QTL test statistic for a 2-QTL model.
10.1186/s12711-016-0199-8 Test statistic of the linkage analysis on BTA20 for the half-sib family sire 2. Dilu: level of coat colour dilution in pigmented areas, HLV: level of hair lengths variation between pigmented and unpigmented coat, HS: hair structure, HD: hair density, RT: RTS classification phenotype.
10.1186/s12711-016-0199-8 Manhattan plots of a whole-genome association study for the traits: hair length variation (HLV), hair structure (HS), hair density (HD) and coat colour dilution (Dilu) in the linear mixed model and for coat colour dilution (Dilu) and hair length variation (HLV) in the case–control design. The upper horizontal line represents the genome-wide significance threshold q = 0.05, the lower horizontal line represents the nominal *p* value = 0.05. Only F_1_, F_2_ and BC individuals with a *E* ^D^/*** and *Dc/dc* + genotype at the *extension* and the *dilution* locus were included.
10.1186/s12711-016-0199-8 Genotypes of wild type and RTS-affected F_2_ individuals for SNP BTA-74302-no-rs. F_2__wt: F_2_ individual with wild type phenotype and F_2__RT: F_2_ individual with RTS phenotype.
10.1186/s12711-016-0199-8 Genes in the region between 10 and 25 Mb on BTA5 that were expressed in the pigmented skin transcriptome of RTS-affected and wild type animals.
