# Supplementary material for: Epistatic interactions between at least three loci determine the “rat-tail” phenotype in cattle
Source: Genet Sel Evol. 2016 Mar 31;48:26. doi: 10.1186/s12711-016-0199-8 (PMC4818457; doi:10.1186/s12711-016-0199-8)

Hair structure (HS), scores 1 – 6

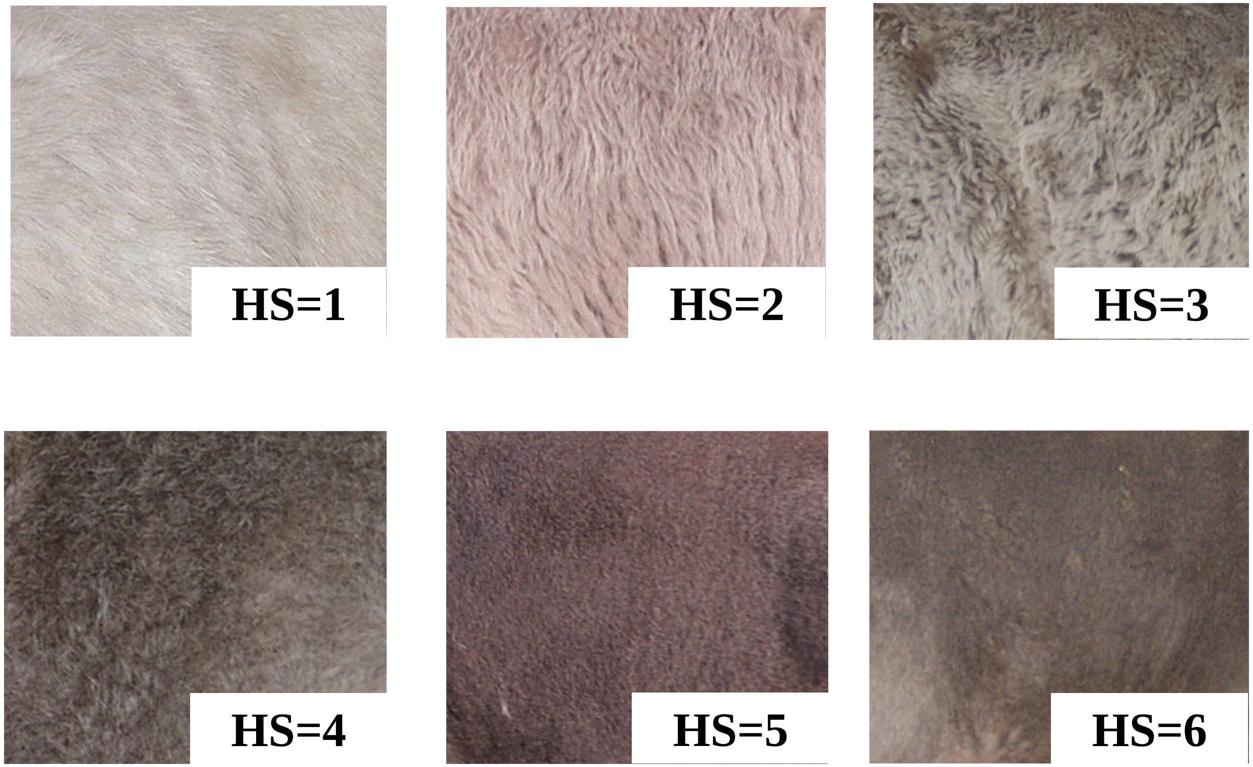

Hair density (HD), scores 1 – 4

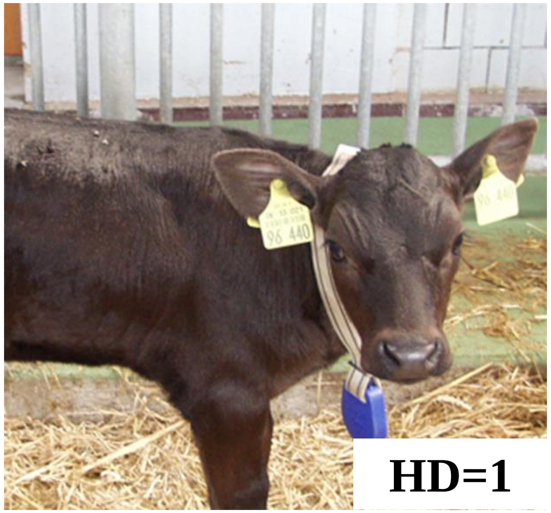

HD=1

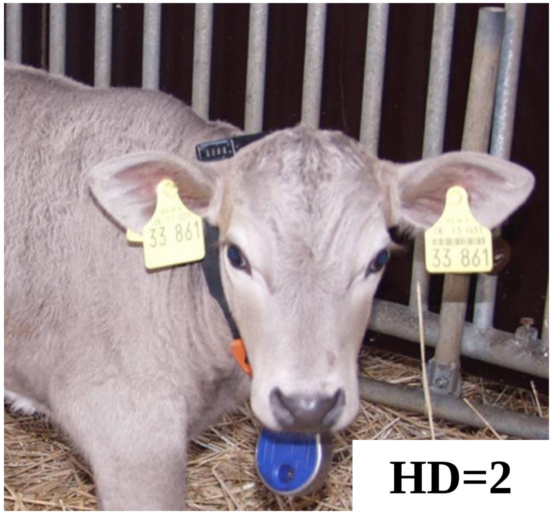

HD=2

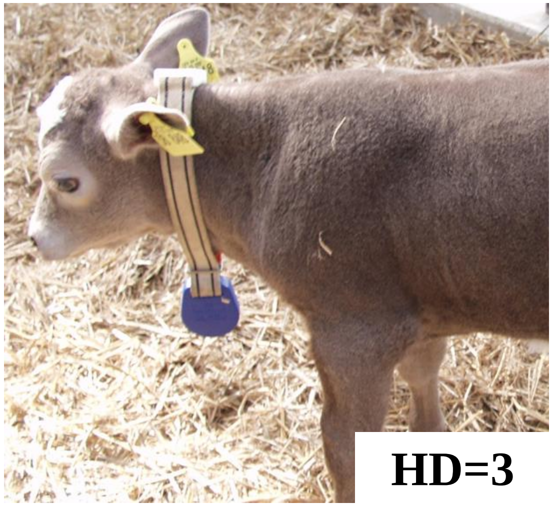

HD=3

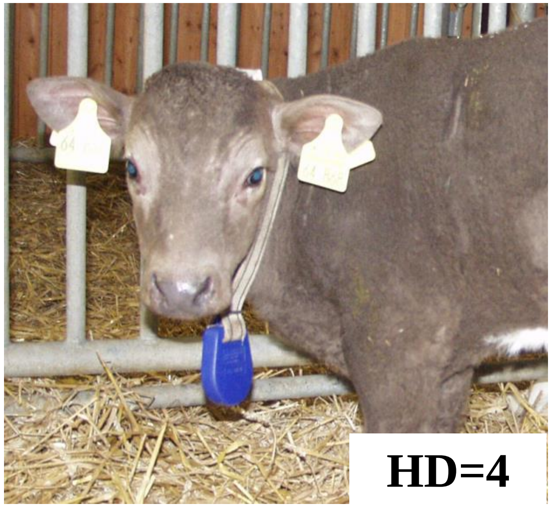

HD=4

Hair length variations (HLV), scores 1 – 4

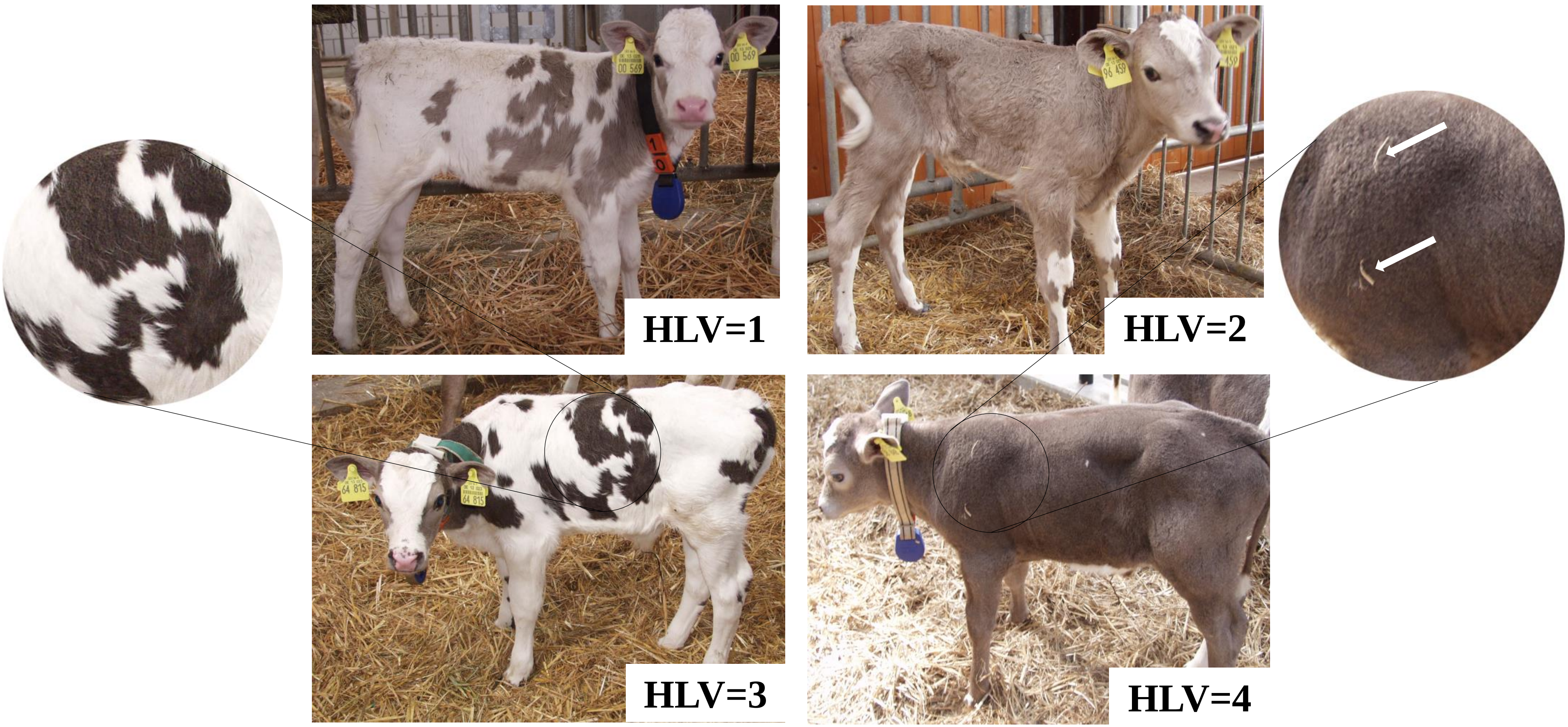

Overall „rat-tail“ phenotype (RT), scores 1 – 4

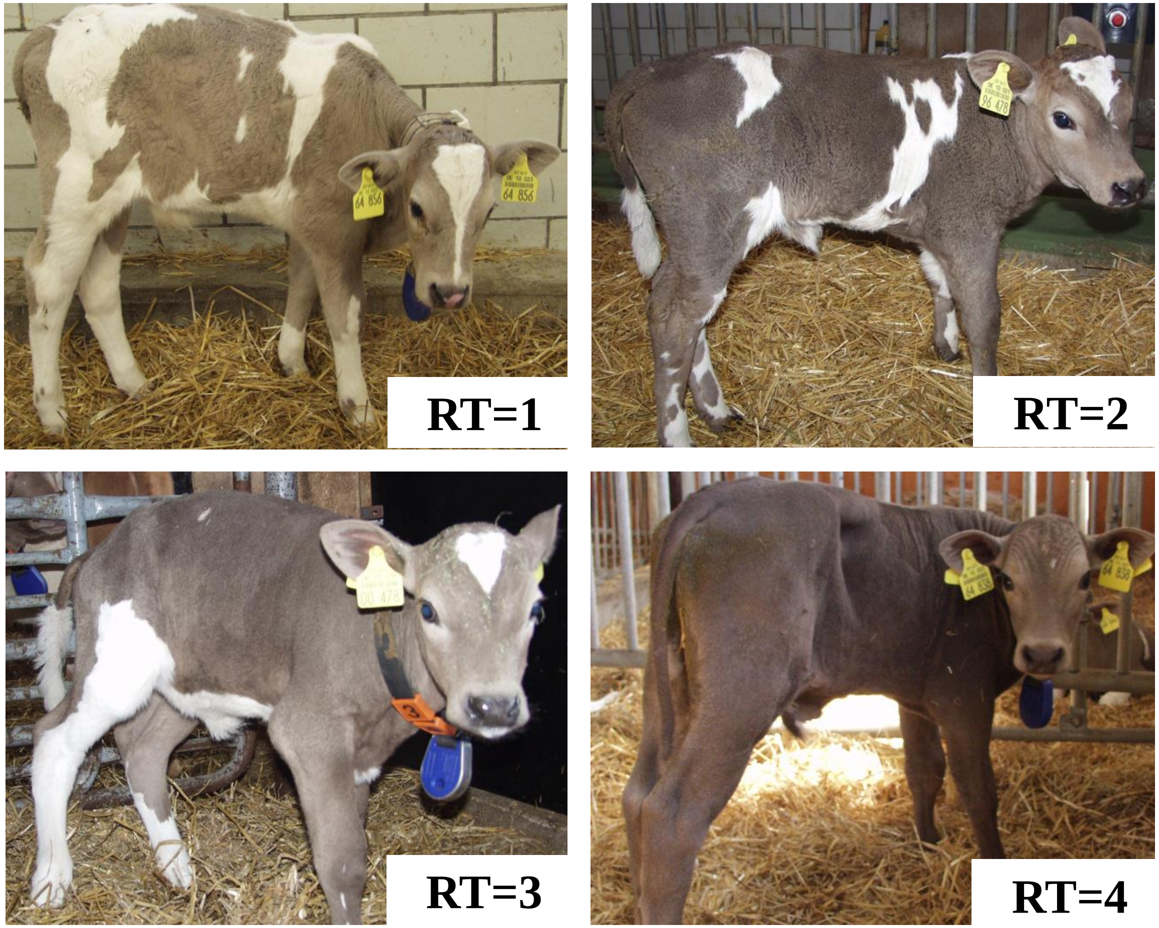

Eumelanic coat colour dilution, scores 1 – 4

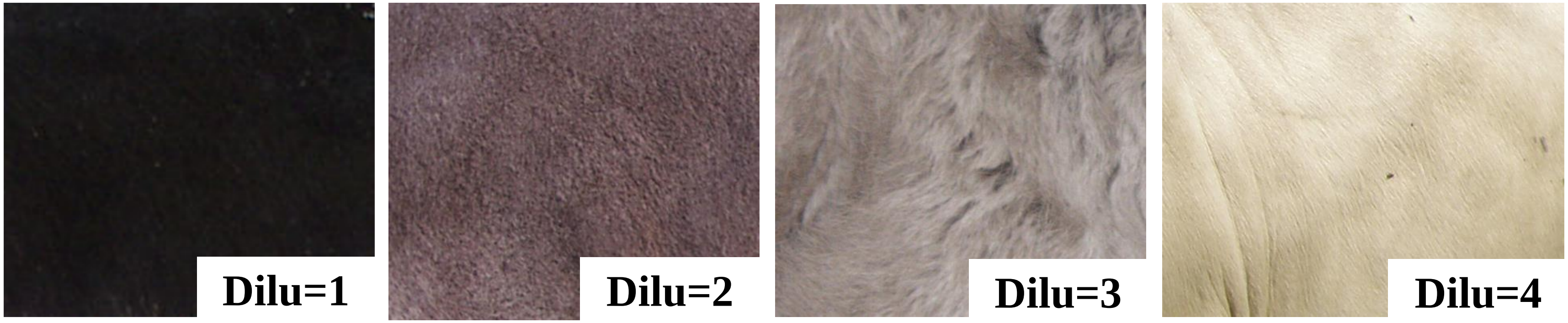

Category 5 – pheomelanic coat colour dilution, scores 1 – 4

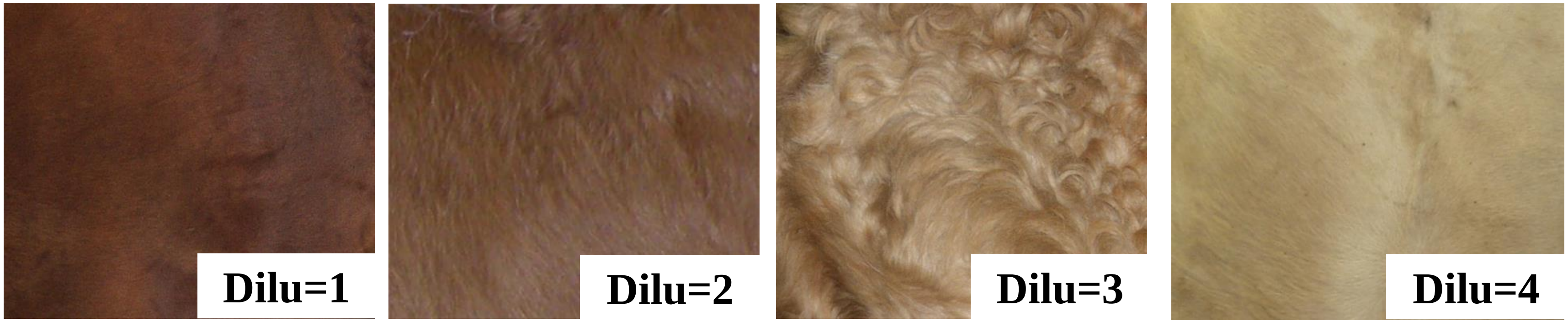

Supplement: Supplementary file 1 — 10.1186/s12711-016-0199-8 Phenotype scoring of RTS associated hair conformation and pigmentation traits. Phenotypes and corresponding scores for HS: HS 1: wild type, straight hair, HS 2: hair with fluffy, smooth structure, HS 3: curly hair with a mildly frizzy/wiry structure, HS 4: coarse, frizzy/wiry hair, HS 5: very coarse/wiry hair, HS 6: extremely coarse/wiry HS. Phenotypes and corresponding scores for hair density (HD): HD 1: wild type, full, thick, long hair covering the whole body as well as in the ears and around the eyes, HD 2: scarce hair at the trunk and/or few, short hair in the ears and around the eyes, HD 3: short and sparse hair in the ears and around the eyes as well as thin coat, HD 4: without eye lashes and/or hair in the ears and with very thin hair coat and/or bald areas. Phenotypes and corresponding scores for hair length variations between pigmented and unpigmented areas of spotted animals (HLV): HLV 1: wild type, equally long hair in pigmented and unpigmented sections. HLV 2: pigmented hair mildly shorter than unpigmented hair, HLV 3: pigmented hair moderately shorter than unpigmented hair, HLV 4: pigmented hair extremely shorter than unpigmented hair. Animals without white spotting marks were scored according to their phenotype for RT. Phenotypes and corresponding scores for RTS classification phenotype: RT 1: wild type without any RTS-associated hair conformation malformation, RT 2: mild HLV and/or short hair in ears and short, thin eye lashes, reduced tail switch. RT 3: sparse, coarse hair, moderate HLV, few hairs in ears/around eyes and/or few hair in eye lashes, tail switch only rudimentary. RT 4: very sparse coat, extreme HLV, scarce/wiry hair in pigmented areas, no hair in ears, barely/no eyelashes, extremely visible skin folds at face/throat, bald areas surrounding the eyes/in the ears, missing tail switch. Status of tail switch was only informative, if the end of the tail was pigmented. Phenotypes and corresponding scores for coat col [file 12711_2016_199_MOESM1_ESM.pdf]
