# Supplementary figures and images for: Epistatic interactions between at least three loci determine the “rat-tail” phenotype in cattle
Source: Genet Sel Evol. 2016 Mar 31;48:26. doi: 10.1186/s12711-016-0199-8 (PMC4818457; doi:10.1186/s12711-016-0199-8)

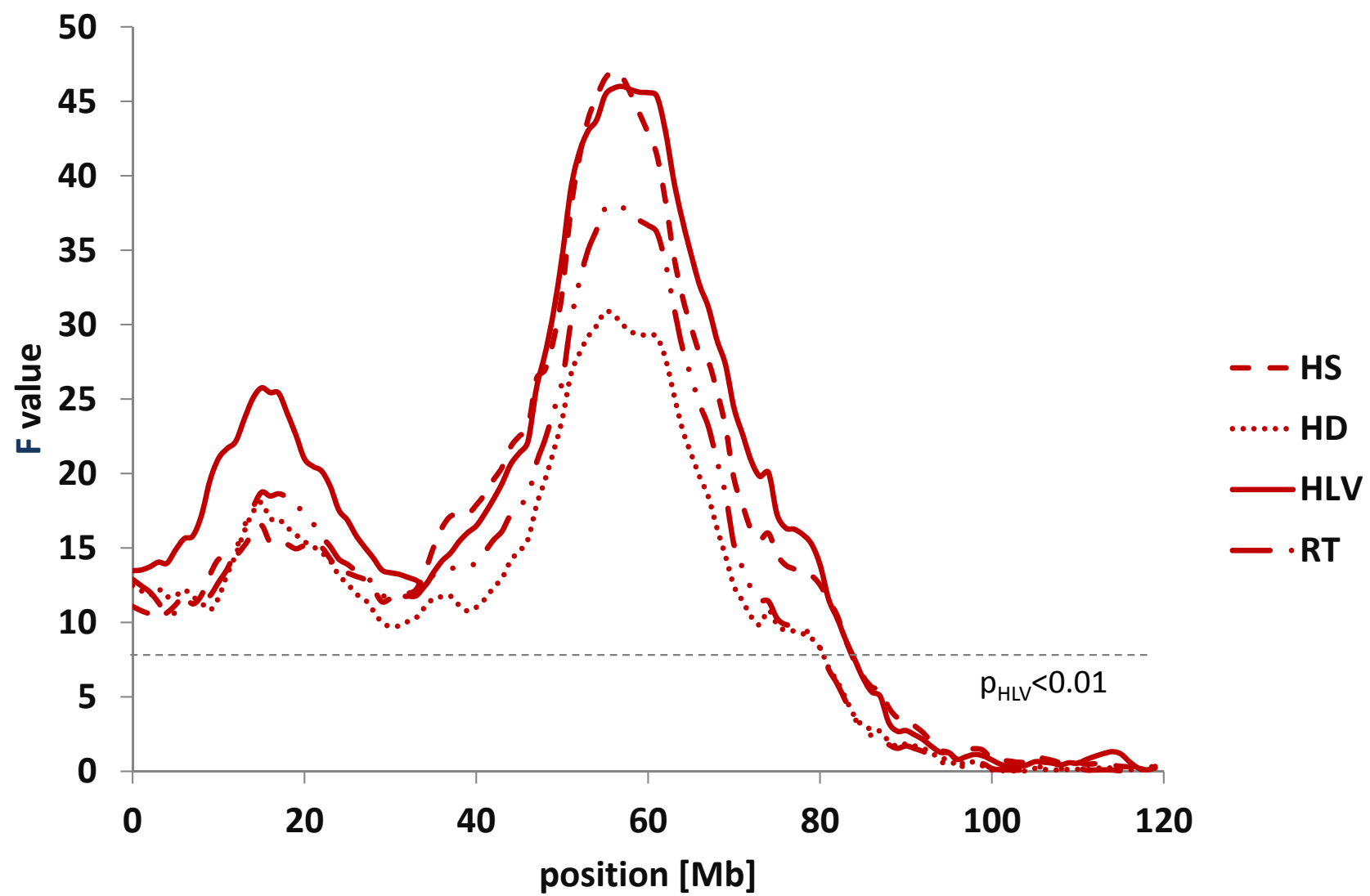

Supplement: Supplementary file 2 — 10.1186/s12711-016-0199-8 Test statistic for hair conformation and pigmentation traits on BTA5 from the F2 full sib design fitting sex as fixed effect in an additive-dominant model. HLV: level of hair length variation between pigmented and unpigmented body areas, HS: hair structure, HD: hair density, RT: RTS classification phenotype. The test statistic for Dilu is not shown due to excessively high F values (>500), which exceed the scale of the y axis. [file 12711_2016_199_MOESM2_ESM.pdf]

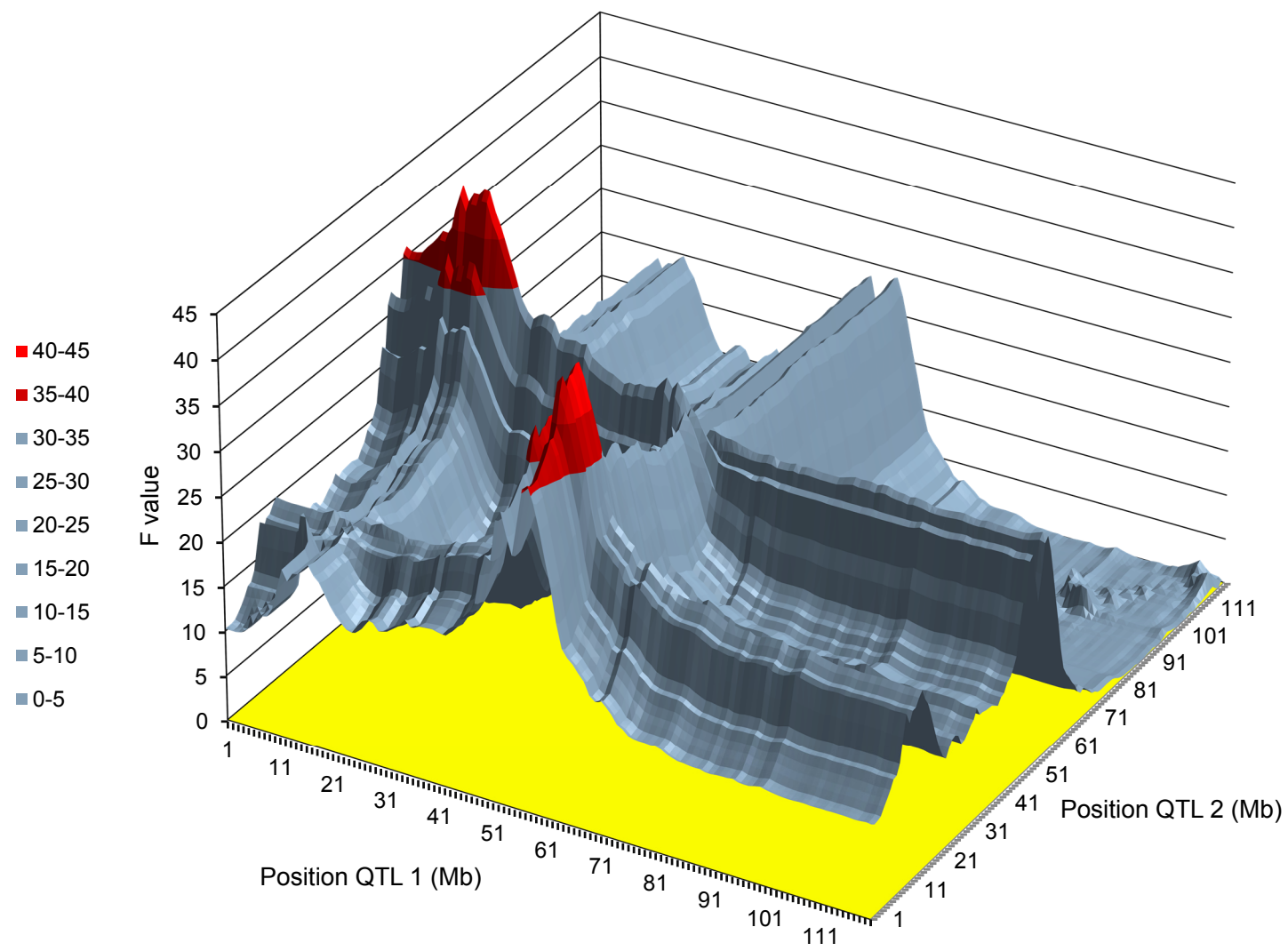

Supplement: Supplementary file 3 — 10.1186/s12711-016-0199-8 Contour plot showing the test statistic for coat colour dilution (Dilu) on BTA5 for a 2-QTL model in a F2 full sib design. The x and y axes represent chromosomal positions for QTL1 and QTL2, respectively. The z axis indicates the F value of the QTL test statistic for a 2-QTL model. [file 12711_2016_199_MOESM3_ESM.pdf]

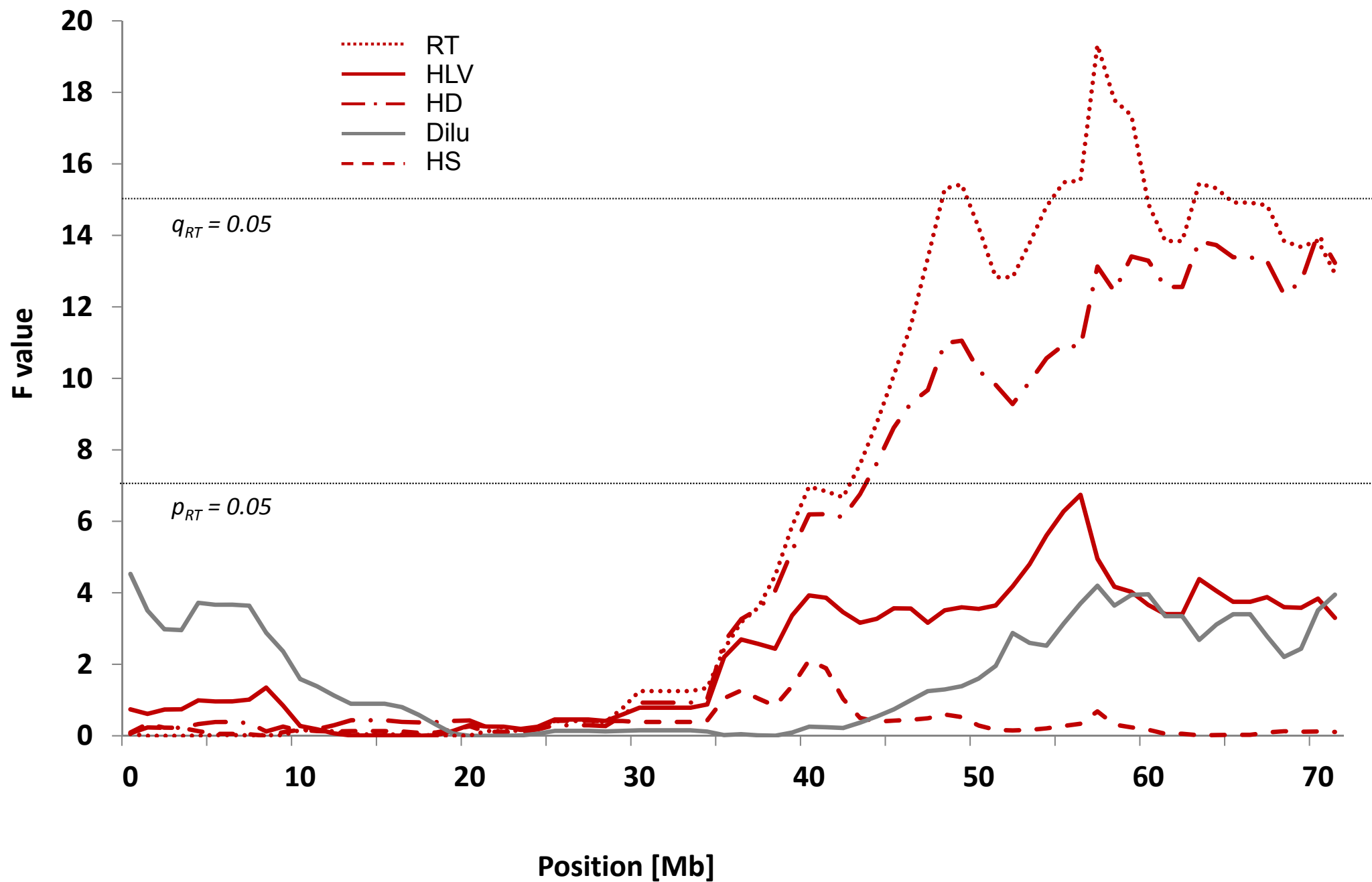

Supplement: Supplementary file 4 — 10.1186/s12711-016-0199-8 Test statistic of the linkage analysis on BTA20 for the half-sib family sire 2. Dilu: level of coat colour dilution in pigmented areas, HLV: level of hair lengths variation between pigmented and unpigmented coat, HS: hair structure, HD: hair density, RT: RTS classification phenotype. [file 12711_2016_199_MOESM4_ESM.pdf]
