# Supplementary material for: Epistatic interactions between at least three loci determine the “rat-tail” phenotype in cattle
Source: Genet Sel Evol. 2016 Mar 31;48:26. doi: 10.1186/s12711-016-0199-8 (PMC4818457; doi:10.1186/s12711-016-0199-8)

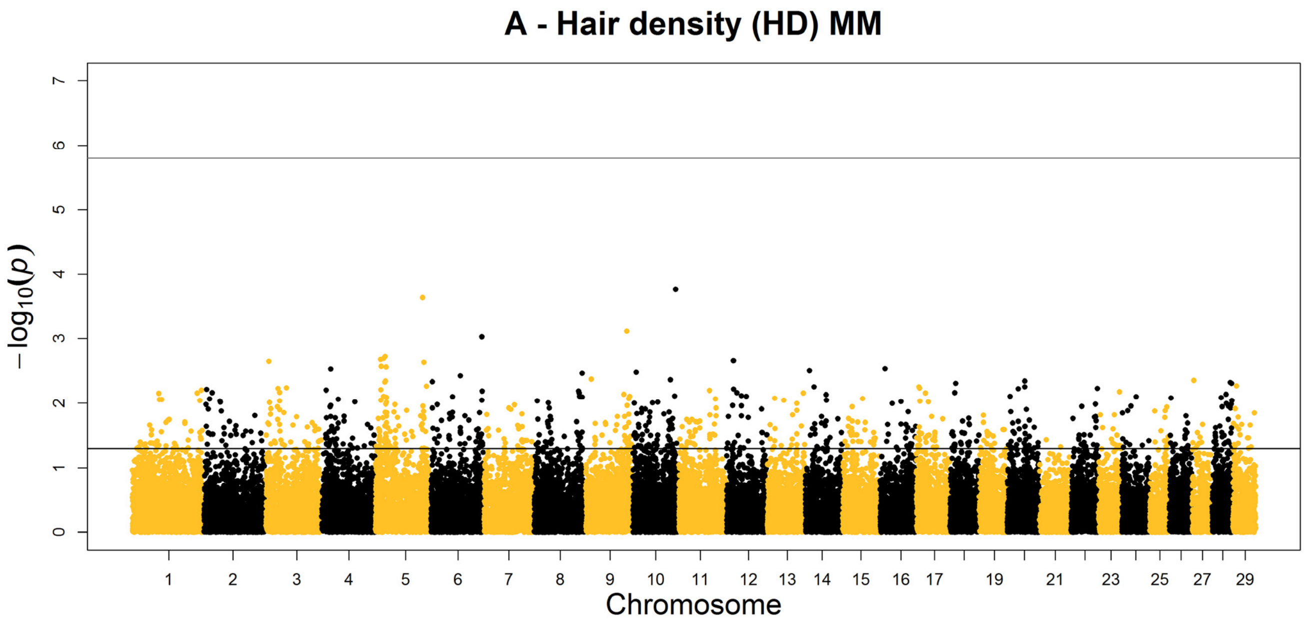
A - Hair density (HD) MM
$-\log_{10}(p)$
0
1
2
3
4
5
6
7
1 2 3 4 5 6 7 8 9 10 11 12 13 14 15 16 17 19 21 23 25 27 29
Chromosome

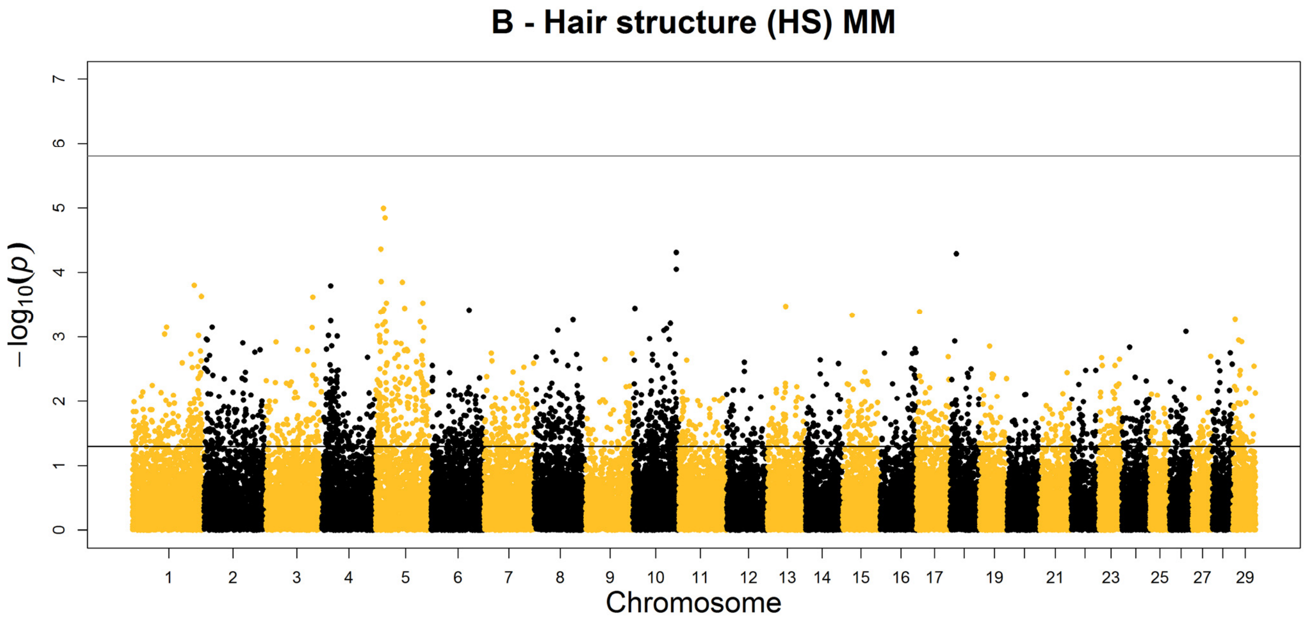
B - Hair structure (HS) MM
$-\log_{10}(p)$
0
1
2
3
4
5
6
7
1 2 3 4 5 6 7 8 9 10 11 12 13 14 15 16 17 19 21 23 25 27 29
Chromosome

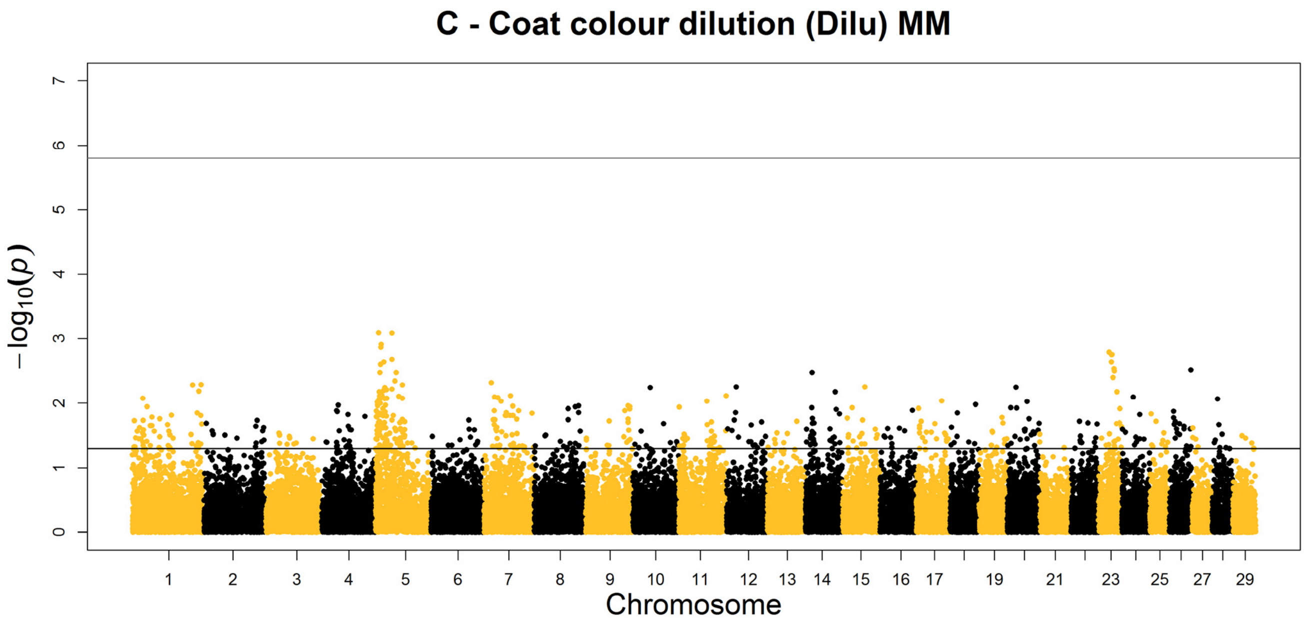
C - Coat colour dilution (Dilu) MM
−log10(p)
0
1
2
3
4
5
6
7
1 2 3 4 5 6 7 8 9 10 11 12 13 14 15 16 17 19 21 23 25 27 29
Chromosome

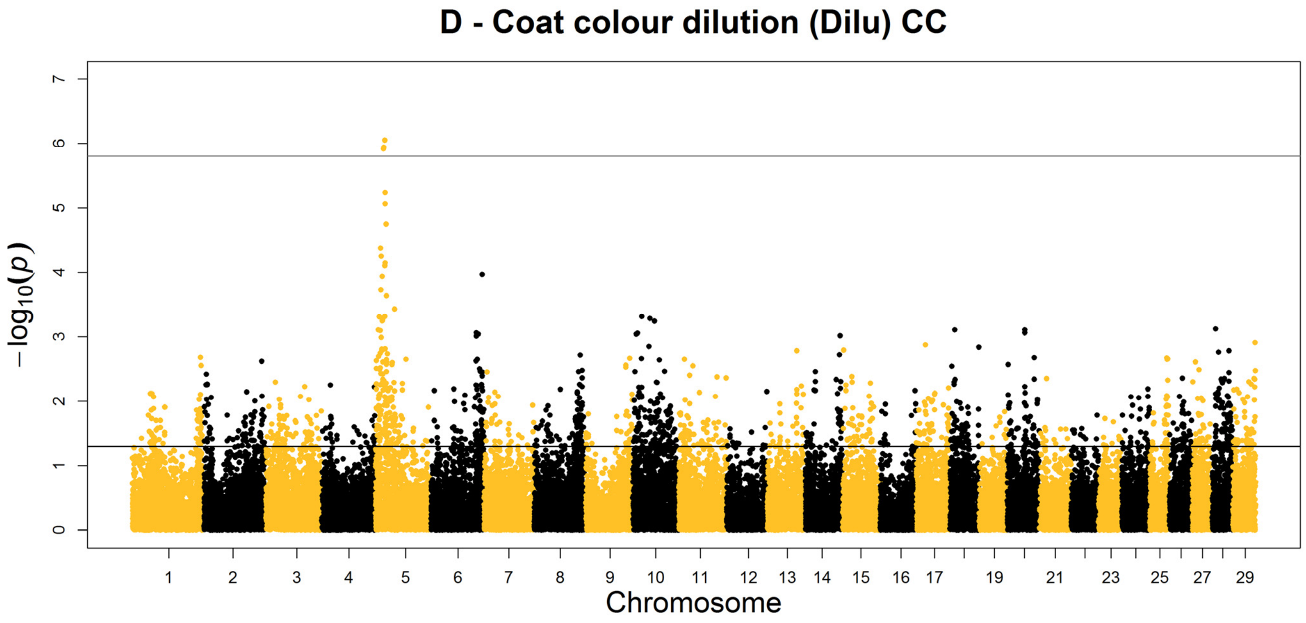
D - Coat colour dilution (Dilu) CC
−log10(p)
0
1
2
3
4
5
6
7
1 2 3 4 5 6 7 8 9 10 11 12 13 14 15 16 17 19 21 23 25 27 29
Chromosome

E - Hair length variation (HLV) MM

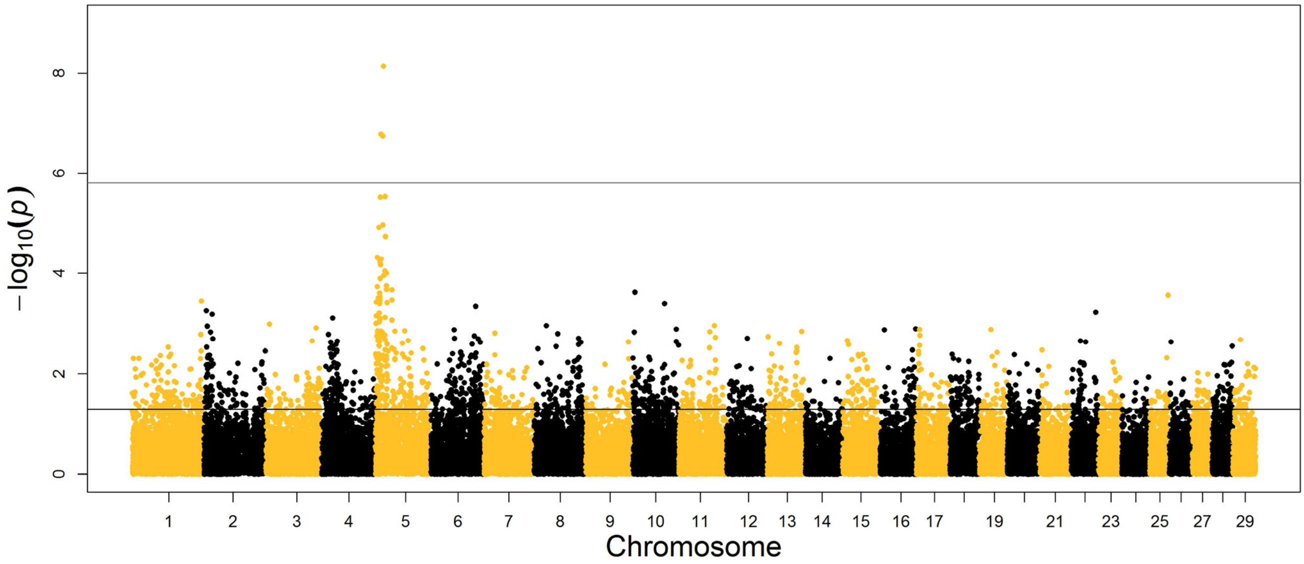

F - Hair length variation (HLV) CC

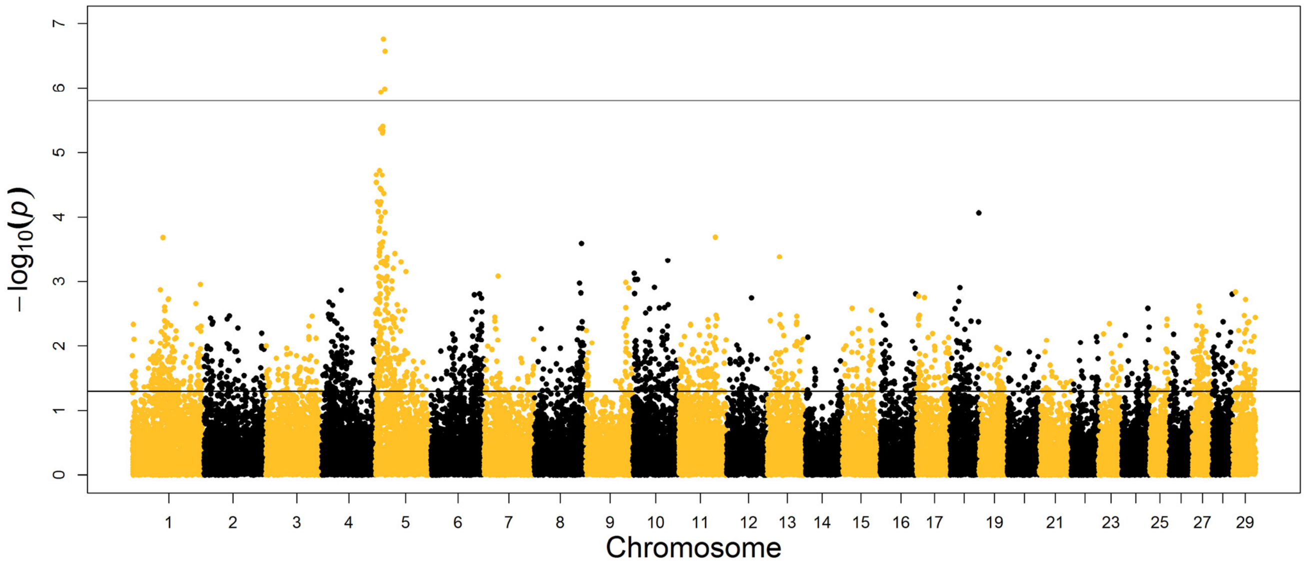

Supplement: Supplementary file 5 — 10.1186/s12711-016-0199-8 Manhattan plots of a whole-genome association study for the traits: hair length variation (HLV), hair structure (HS), hair density (HD) and coat colour dilution (Dilu) in the linear mixed model and for coat colour dilution (Dilu) and hair length variation (HLV) in the case–control design. The upper horizontal line represents the genome-wide significance threshold q = 0.05, the lower horizontal line represents the nominal p value = 0.05. Only F1, F2 and BC individuals with a E D/* and Dc/dc + genotype at the extension and the dilution locus were included. [file 12711_2016_199_MOESM5_ESM.pdf]
