# Supplementary material for: Epistatic interactions between at least three loci determine the “rat-tail” phenotype in cattle
Source: Genet Sel Evol. 2016 Mar 31;48:26. doi: 10.1186/s12711-016-0199-8 (PMC4818457; doi:10.1186/s12711-016-0199-8)
Supplement: Supplementary file 6 — 10.1186/s12711-016-0199-8 Genotypes of wild type and RTS-affected F2 individuals for SNP BTA-74302-no-rs. F2_wt: F2 individual with wild type phenotype and F2_RT: F2 individual with RTS phenotype. [file 12711_2016_199_MOESM6_ESM.pdf]

Group	Individual	Allele1	Allele2
F_2_RT	30105	1	2
F_2_RT	40120	2	2
F_2_RT	20410	2	2
F_2_wt	30106	1	2
F_2_wt	41804	1	1
F_2_wt	40112	1	2
F_2_wt	71114	1	2
