# Supplementary material for: Epistatic interactions between at least three loci determine the “rat-tail” phenotype in cattle
Source: Genet Sel Evol. 2016 Mar 31;48:26. doi: 10.1186/s12711-016-0199-8 (PMC4818457; doi:10.1186/s12711-016-0199-8)
Supplement: Supplementary file 7 — 10.1186/s12711-016-0199-8 Genes in the region between 10 and 25 Mb on BTA5 that were expressed in the pigmented skin transcriptome of RTS-affected and wild type animals. [file 12711_2016_199_MOESM7_ESM.pdf]

Additional file 6: Tab. S1: Genes in the region 10 - 25 Mb on BTA5 found to be expressed in the pigmented skin transcriptome of RTS-affected and wild type animals

Gene	Name	Position [Mb] [1]	Number of exons[2]			Comment
			RTS A	wild type	RTS B, RTS C	
MYF6	myogenic factor 6 (herculin)	10,330,470	3	3	3	
LIN7A	lin-7 homolog A (C. elegans)	10,566,010	6	6	6	
ACSS3	acyl-CoA synthetase short-chain family member 3	10,731,243	16	16	16	
CCDC59	coiled-coil domain containing 59	11,378,864	4	4	4	
METTL25	methyltransferase like 25	12,007,564	9	9	9	
TMTC2	transmembrane and tetratricopeptide repeat containing 2	12,290,944	13	13	13	
LOC781108	PRKR-interacting protein 1 pseudogene	13,756,337	1	1	1	
SLC6A15	solute carrier family 6 (neutral amino acid transporter), member 15	14,572,179	12	12	12	
ALX1	ALX homeobox 1	14,991,697	4	4	4	
RASSF9	Ras association (RalGDS/AF-6) domain family (N-terminal) member 9	15,546,053	2	2	2	
C5H12orf29	chromosome 5 open reading frame, human C12orf29	17,892,374	7	7	7	
CEP290	centrosomal protein 290kDa	17,998,514	55;56	55;56	55;56	
TMTC3	transmembrane and tetratricopeptide repeat containing 3	17,998,756	14;13	14;13	14;13	
KITLG	KIT ligand	18,420,198	9;8	9;8	9;8	Additional exon 1
DUSP6	dual specificity phosphatase 6	19,280,061	3	3	3	
POC1B	POC1 centriolar protein B	19,462,615	12*	12;10	12	No RTS-associated splincing
GALNT4	UDP-N-acetyl-alpha-D-galactosamine:polypeptide N-acetylgalactosaminyltransferase 4 (GalNAc-T4)	19,461,415	1	1	1	
ATP2B1	ATPase, Ca++ transporting, plasma membrane 1	19,670,561	21	21	21	2 transcription start sites, no RTS-associated splicing

KERA	keratocan	21,003,034	3	3	3	
LUM	lumican	21,047,145	4;3	4;3	4;3	2 transcription start sites, additional exon 1
DCN	decorin	21,119,087	8	8	8	2 transcription start sites
BTG1	B-cell translocation gene 1, anti-proliferative	22,088,782	2	2	2	
LOC784883	putative gamma-taxilin-like protein CYorf15B	22,526,151	3	3	3	
LOC519417	pleckstrin homology domain-containing family G member 7	22,719,202	16	16	16	
EEA1	early endosome antigen 1	22,932,444	30*;29*; 28	30*;29*; 28	30*;29*;28	2 previously unknown additional exons
NUDT4	nudix (nucleoside diphosphate linked moiety X)-type motif 4	23,348,044	5	5	5	
UBE2N	ubiquitin carrier protein N|ubiquitin-conjugating enzyme E2 N|ubiquitin-protein ligase N	23,411,877	4	4	4	
MRPL42	mitochondrial ribosomal protein L42	23,426,113	7,7*,6	7,7*,6	7,7*,6	
SOCS2	suppressor of cytokine signaling 2	23,524,065	3	3	3	
CRADD	CASP2 and RIPK1 domain containing adaptor with death domain	23,629,05	3	3	3	additional exon 3 compared to annotation 104
PLXNC1	plexin C1	24,072,234	31;30	31;30	31;30	
CEP83	centrosomal protein 83kDa	24,374,925	17;16	17;16	17;16	
LOC100848598	uncharacterized LOC100848598	24,380,708	3;2	3;2	3;2	
TMCC3	transmembrane and coiled-coil domain family 3	24,537,335	4	4	4	
NDUFA12	NADH dehydrogenase (ubiquinone) 1 alpha subcomplex, 12	24,873,028	4;3	4;3	4;3	
NR2C1	nuclear receptor subfamily 2, group C, member 1	24,922,158	14	14	14	
FGD6	FYVE, RhoGEF and PH domain containing 6	25,031,325	21, 20*	21	21	

[1]First base of exon 1 according to *Bos taurus* genome assembly UMD3.1.1.

[2]Number of exons in pigmented skin of three individuals (RTS A, RTS B, RTS C) with “rat-tail” phenotype and an individual with wild type phenotype.

[*]Splice variants compared annotation release 104 of the *Bos taurus* genome assembly
